# Astrocytes Sustain Circadian Oscillation and Bidirectionally Determine Circadian Period, But Do Not Regulate Circadian Phase in the Suprachiasmatic Nucleus

**DOI:** 10.1523/JNEUROSCI.2337-21.2022

**Published:** 2022-07-13

**Authors:** Andrew P. Patton, Nicola J. Smyllie, Johanna E. Chesham, Michael H. Hastings

**Affiliations:** MRC Laboratory of Molecular Biology, Cambridge CB2 0QH, United Kingdom

**Keywords:** calcium, chemogenetics, cryptochrome, fluorocitrate, glia, neuron

## Abstract

The suprachiasmatic nucleus (SCN) is the master circadian clock of mammals, generating and transmitting an internal representation of environmental time that is produced by the cell-autonomous transcriptional/post-translational feedback loops (TTFLs) of the 10,000 neurons and 3500 glial cells. Recently, we showed that TTFL function in SCN astrocytes alone is sufficient to drive circadian timekeeping and behavior, raising questions about the respective contributions of astrocytes and neurons within the SCN circuit. We compared their relative roles in circadian timekeeping in mouse SCN explants, of either sex. Treatment with the glial-specific toxin fluorocitrate revealed a requirement for metabolically competent astrocytes for circuit-level timekeeping. Recombinase-mediated genetically complemented Cryptochrome (Cry) proteins in Cry1-deficient and/or Cry2-deficient SCNs were used to compare the influence of the TTFLs of neurons or astrocytes in the initiation of *de novo* oscillation or in pacemaking. While neurons and astrocytes both initiated *de novo* oscillation and lengthened the period equally, their kinetics were different, with astrocytes taking twice as long. Furthermore, astrocytes could shorten the period, but not as potently as neurons. Chemogenetic manipulation of Gi- and Gq-coupled signaling pathways in neurons acutely advanced or delayed the ensemble phase, respectively. In contrast, comparable manipulations in astrocytes were without effect. Thus, astrocytes can initiate SCN rhythms and bidirectionally control the SCN period, albeit with lower potency than neurons. Nevertheless, their activation does not influence the SCN phase. The emergent SCN properties of high-amplitude oscillation, initiation of rhythmicity, pacemaking, and phase are differentially regulated: astrocytes and neurons sustain the ongoing oscillation, but its phase is determined by neurons.

**SIGNIFICANCE STATEMENT** The hypothalamic suprachiasmatic nucleus (SCN) encodes and disseminates time-of-day information to allow mammals to adapt their physiology to daily environmental cycles. Recent investigations have revealed a role for astrocytes, in addition to neurons, in the regulation of this rhythm. Using pharmacology, genetic complementation, and chemogenetics, we compared the abilities of neurons and astrocytes in determining the emergent SCN properties of high-amplitude oscillation, initiation of rhythmicity, pacemaking, and determination of phase. These findings parameterize the circadian properties of the astrocyte population in the SCN and reveal the types of circadian information that astrocytes and neurons can contribute within their heterogeneous cellular network.

## Introduction

Mammalian behavioral and physiological circadian rhythms are orchestrated by the hypothalamic suprachiasmatic nucleus (SCN), which is synchronized to environmental time via the retinohypothalamic tract (Reppert and Weaver, 2002; Patton and Hastings, 2018). This retinal innervation is not, however, required for the SCN to create and broadcast a representation of environmental time: SCN timekeeping in isolation *ex vivo* is robust and can persist with precision nearly indefinitely, sustaining rhythms ranging between <16 h and >42 h across a range of genetic and pharmacological manipulations (Patton et al., 2016). This robustness arises from strong, presumably reciprocal, network interactions between the ∼10,000 neurons and 3500 glia, including astrocytes (Hastings et al., 2018).

At its core, timekeeping in the majority of cells in the body is directed by the activity of a transcriptional/post-translational feedback loop (TTFL) whereby Period (Per) and Cryptochrome (Cry) proteins act on the transcription factors CLOCK and BMAL1 to repress their own transcription (Partch et al., 2014). This generates a rhythmic alternation of activation and repression, over the course of ∼24 h, of the *Per* and *Cry* genes and their transcriptional targets. It is this TTFL-based oscillation, welded into the strong network architecture of the SCN that produces robust timekeeping at the circuit level. This is characterized by emergent properties of high-amplitude precise oscillation, tightly defined ensemble period and phase, and spatially complex cellular synchrony (Hastings et al., 2018; Patton et al., 2020).

Until recently, the GABAergic and neuropeptidergic neurons were thought to be the major SCN timekeeping component, becoming electrically and metabolically active in the middle of circadian day and quiescent during circadian night (Colwell, 2011; Brancaccio et al., 2017). Consistent with this view, recent intersectional genetic approaches (for review, see Patton et al., 2020) have revealed that SCN neurons together, as a whole, are sufficient for maintaining and initiating circadian rhythms in the SCN *ex vivo* and *in vivo* (Lee et al., 2015; Maywood et al., 2018; Brancaccio et al., 2019) as well as setting the relative phase (Jones et al., 2015) and determining the dynamics of the ongoing oscillation (Brancaccio et al., 2013; Lee et al., 2015).

Alongside these approaches in neurons, recent studies have also revealed astrocytes as active participants in the SCN network. Astrocytes display robust rhythms in TTFL function (Tso et al., 2017; Brancaccio et al., 2019) and cytosolic calcium ([Ca^2+^]_i_; Brancaccio et al., 2017), sitting in antiphase to neuronal rhythms (Brancaccio et al., 2017, 2019). Additionally, the astrocytic TTFL clock is sufficient to determine SCN and behavioral period (Brancaccio et al., 2017; Tso et al., 2017) and even initiate rhythmicity in an otherwise “clockless” SCN, driving neuronal [Ca^2+^]_i_ rhythms and behavioral rhythms (Brancaccio et al., 2019). In contrast to this initiation, disruption of the astrocytic TTFL lengthens the behavioral and *ex vivo* SCN periods (Barca-Mayo et al., 2017; Tso et al., 2017). Furthermore, manipulation of a putative astrocyte-neuron adenosine/cannabinoid signaling axis can induce phase shifts during the circadian day (Hablitz et al., 2020). Astrocytes can therefore impose their circadian state on SCN neurons and the whole animal, potentially via three mechanisms that modulate GABAergic signaling as follows: active release of glutamate by astrocytes (Brancaccio et al., 2017); active GABA uptake contributing to a vasoactive intestinal polypeptide (VIP)/GABA balance (Barca-Mayo et al., 2017); or astrocytic adenosine release (Hablitz et al., 2020).

These findings raise the question of the relative potency of astrocytes, compared with neurons, in determining the circadian properties of the SCN circuit. Using pharmacology, calcium imaging, intersectional genetics, and chemogenetic manipulation, we therefore compared the abilities and strengths of astrocytes and neurons to determine the following emergent, ensemble timekeeping properties of the SCN: high-amplitude oscillation, rhythm initiation, period, and phase. We show that, whereas astrocytes are powerful regulators of the ongoing, steady-state SCN oscillation, their activation does not contribute to resetting the ensemble oscillation to a new phase: entrainment is mediated by neuronal activity.

## Materials and Methods

### Animals.

All experiments were performed in accordance with the UK Animals (Scientific Procedures) Act of 1986, with local ethical approval (MRC LMB AWERB). Per2::Luciferase mice were supplied by J.S. Takahashi (University of Texas Southwestern Medical Center, Dallas, TX; Yoo et al., 2004). Cry1-null, Cry2-null, and Cry1,2-null (Cry-null) animals were derived from founders supplied by G. van der Horst (Erasmus University Medical Center, Rotterdam, The Netherlands; van der Horst et al., 1999). Cry1-null, Cry2-null, and Cry-null mice were crossed to the Per2::Luciferase line in-house. All lines were maintained on a C57BL/6J background.

### AAVs and molecular biology.

For Cry-complementation experiments, Cre-conditional Cry1::EGFP (*pCry1*-DIO.Cry1::EGFP) was packaged by Penn Vector Core as an AAV1 serotype from a plasmid backbone produced in-house (Brancaccio et al., 2019). The Cre-conditional Cry2::EGFP (*pCry2*-DIO.CRY2::EGFP) adeno-associated virus (AAV) was generated by first modifying AAV.*pCry1*(min)-mCry2::EGFP (Edwards et al., 2016) through replacement of the minimal *pCry1* promoter with a minimal *pCry2* promoter (Smyllie et al., 2022). The coding sequence was inverted, and double-inverted orientation LoxP sites were added to create a Cre-inducible version before being packaged as the AAV1 serotype (Vector Builder). pZac2.1.gfaABC1D.cyto-GCaMP6f (*GFAP*-cytoGCaMP6f) was a gift from Baljit Khakh [University of California, Los Angeles, Los Angeles, CA; viral prep #52 925-AAV5, Addgene (RRID:Addgene_52925); Haustein et al., 2014]. The chemogenetic constructs hM3D(Gq) and hM4D(Gi) under the control of either the *hSyn* or *GFAP* promoters were a gift from Bryan Roth [University of North Carolina (UNC), Chapel Hill, NC] and supplied as follows: pAAV.GFAP.hM3D(Gq)::mCherry.WPRE.bGHpA (*GFAP*-hM3Dq::mCherry; viral prep #50478-AAV5, Addgene; RRID:Addgene_50478); pAAV.GFAP.hM4d(Gi)::mCherry.WPRE.bGHpA (*GFAP*-hM4Di::mCherry; viral prep #50479-AAV5, Addgene; RRID:Addgene_50479); pAAV.hSyn.hM3D(Gq)::mCherry.WPRE.bGHpA (*Syn*-hM3Dq::mCherry; viral prep #50474-AAV8, Addgene; RRID:Addgene_50474); and pAAV.hSyn.hM4d(Gi)::mCherry.WPRE.bGHpA (*Syn*-hM4Di::mCherry; viral prep #50475-AAV8, Addgene; RRID:Addgene_50475). Cre-recombinase AAVs (*hSyn*-mCherry::Cre and *GFAP*-mCherry::Cre) were AAV8 serotype (UNC Vector Core). pAAV.EF1a.DIO.EYFP (*EF1a*-DIO.EYFP) was a gift from Karl Deisseroth (Stanford University, Stanford, CA; viral prep #27056-AAV1, Addgene; RRID:Addgene_27056).

### Organotypic slice preparation and AAV transduction.

Mice [postnatal day 10 (P10) to P12] of either sex were killed according to local and UK Home Office rules, brain were removed, and the SCNs dissected before being sliced coronally and cultured as an explant via the interface method for 1 week (Hastings et al., 2005). The culture medium was then changed, and slices were transduced with 1 µl of AAV (>1 × 10^12^ genome copies/ml in PBS) applied directly to the top of the slice. Slices were then left for a further week before the medium was changed. Where slices received more than one transduction, this was performed serially with 24 h between medium change (MC) and AAV application. Successful transduction was confirmed by imaging of the encoded fluorescent reporter.

### Real-time bioluminescent and fluorescent imaging.

Bioluminescence was monitored in real-time in customized light-tight incubators equipped with photon-multiplier tubes (PMTs; catalog #H9319-11 photon counting head, Hamamatsu), before data were binned at 6 min intervals for export. SCN slices were maintained in HEPES-buffered medium containing DMEM, supplemented with Glutamax, penicillin/streptomycin, FCS, B27, and luciferin in dishes sealed with glass coverslips, as previously described (Patton et al., 2016).

For combined circadian fluorescent/bioluminescent recordings, SCN slices were maintained in sealed, glass-bottomed imaging dishes (MATTEK) with the same medium as in PMT recordings and imaged on an LV200 Bioluminescence Imaging System (Olympus; Brancaccio et al., 2013). Dependent on the experimental configuration, bioluminescence was acquired for between 9.5 and 29.5 min, while simultaneous fluorescence acquisition was set at 100 ms [enhanced yellow fluorescent protein (EYFP), mCherry] or 250 ms (GCaMP6f). The acquisition intervals for combined circadian imaging were 30 min.

For calcium imaging of SCN slices to test acute designer receptor exclusively activated by designer drugs (DREADD) activation, vehicle (Veh; PBS), or 100 nm clozapine-*N*-oxide (CNO; in PBS) were added as a 1 µl drop directly to the surface of the slice on the heated stage of an LV200 Bioluminescence Imaging System. Because of the rapid kinetics of the response in *GFAP*-hM4Di::mCherry and *GFAP*-cytoGCaMP6f coexpressing slices (see Fig. 4), recordings were made using the GFP channel at an acquisition rate of 5 Hz with the exposure interval set to 100 ms. Because of the comparatively slower kinetics of the response in *GFAP-*hM3Dq::mCherry and *GFAP*-cytoGCaMP6f coexpressing slices (see Fig. 4), recordings were made using the GFP channel at an acquisition rate of 0.5 Hz with the exposure interval set to 100 ms. In both experimental configurations, slices were recorded for a baseline (BL) period of 1 min before either vehicle or CNO were added to the preparation and recording continued for another 5 min. Before and after the experiment, mCherry and bright-field images were taken with exposure times of 100 and 10 ms, respectively.

### Analysis of bioluminescent and fluorescent imaging.

All PMT data were analyzed by BioDARE2 (https://www.biodare2.ed.ac.uk; Moore et al., 2014; Zielinski et al., 2014), using the fast Fourier transform (FFT)-nonlinear least squares method (Maywood et al., 2018) before additional analyses were applied. In the case of rhythmic SCN genotypes, a peak-to-peak method was applied where all the peaks in a recording were identified and the mean time difference between consecutive peaks was taken as the period for that cycle. Where the point of initiation of rhythmicity was determined based on instantaneous period, the waveclock package (Price et al., 2008) for R (R Core Team; https://www.R-project.org/) was used and the period output was used to determine the time point at which period crossed the 25–29 h threshold (i.e., 2 h either side of the expected initiated period identified by BioDARE). This was once the time point was outside the cone of influence (defined as √2 * period) where the reported data may be affected by “edge effects” at the beginnings and ends of the time series. Rhythmicity and arrhythmicity were measured by calculating the autocorrelation of recordings that were detrended by fitting a second-order polynomial function at a lag of 24 h (in the case of wild-type comparisons) or 28 h (in the case of Cry1-competent rhythms). Autocorrelation was calculated in R using the acf command in the base R stats package.

Automated ROI analysis of real-time circadian bioluminescence imaging was performed as described previously (Patton et al., 2016) using the SARFIA package (Dorostkar et al., 2010) in Igor Pro 8 (WaveMetrics). Recordings were represented as a false-colored raster plot. All raster plots were colored using the viridis color scale. Using these data, synchrony was also determined by manual calculation of Rayleigh vectors in Microsoft Excel from circadian parameters determined in BioDARE2 (Patton et al., 2016).

Circadian calcium imaging data were analyzed as described previously (Patton et al., 2020). Briefly, fluorescence images were aligned manually and background subtracted in FIJI (Schindelin et al., 2012) before full *z*-stacks were taken to obtain aggregate fluorescence signals. In the case of circadian imaging, these signals were detrended and, in the fluorocitrate experiments, fluorescence changes were expressed as relative amplitude by dividing the detrended data by the average of the raw signal for that experimental interval. Because of the continual rise in the fluorescence baseline as a function of continuing AAV-mediated expression of the reporter, this was deemed the most appropriate means of normalizing the data to allow comparison between different temporal windows across the entire recording.

For analysis of calcium imaging of acutely manipulated DREADD-expressing Cry-null SCN slices, aggregate signals were taken as described above following alignment and background subtraction, but these recordings did not require detrending. Aggregate recordings were normalized using a (*F* – *F*_0_)/*F*_0_ method where *F*_0_ was designated as the average of the baseline for 1 min before treatment. The peak change in this measurement was calculated by taking the average of the first 30 s immediately following treatment. Finally, network dynamics across the SCN were examined by creating raster plots using automated SARFIA ROI analysis routines, as described above but using the DREADD-mCherry signal to direct ROIs to DREADD-expressing cells.

### Pharmacological treatments.

For fluorocitrate experiments, drug or vehicle were prepared fresh in parallel immediately before treatment (Paulsen et al., 1987). Briefly, a stock solution of fluorocitrate was prepared by dissolving 8 mg of fluorocitrate barium salt in 1 ml of 0.1 m HCl before 2–3 drops of 0.1 m Na_2_SO_4_ were added to the solution to precipitate the barium. Finally, 2 ml of Na_2_HPO_4_ was added, and the solution was centrifuged at 1000 × *g* for 5 min. The supernatant containing fluorocitrate was adjusted to pH 7.4 and then pipetted off and added directly to the medium at a final concentration of 50 µm. For the preparation of vehicle, fluorocitrate barium salt was omitted, and an equal volume of vehicle was added to the medium. Treatments were left *in situ* for up to 5 d before they were washed out by transferring the slice between prewarmed recording medium three times at 5 min intervals.

For chemogenetic experiments, DREADD-expressing slices were recorded in PMTs for at least four cycles to generate a baseline from which the phase of treatment was extrapolated. At the correct phase for treatment, slices affixed to membranes were transferred to prewarmed recording medium containing either 100 nm CNO or 0.01% H_2_O (vehicle). Slices were then left in this medium for 1 h before the treatment was washed out by transferring the slice between prewarmed recording medium three times at 5 min intervals. Finally, the slices were returned to their original recording medium and recorded for at least three further cycles. Each slice was subjected to at least two treatments at the same phase (CNO and vehicle), the order of which was randomized in the *GFAP*-Gq-, *GFAP*-Gi-, and *Syn*-Gi-expressing slices. In the case of *Syn*-Gq, slices were treated with vehicle before being treated with CNO as *Gq*-activation in SCN neurons has been shown previously to perturb ongoing SCN function permanently through a presumed VIPergic mechanism (Brancaccio et al., 2013; Hamnett et al., 2019). All phase shifts were assessed as paired measures where a slice was subjected to vehicle and CNO at a particular phase. Any slice that did not receive both treatments was excluded from analysis. Phase shifts were calculated as described previously (Patton et al., 2020). Briefly, the four cycles of pretreatment were used to calculate the baseline period, which was used to normalize subsequent shifts in circadian time. Phase shifts were calculated as the difference between the actual peaks and the predicted peaks extrapolated forward by the mean baseline period from the peak preceding treatment and expressed as the mean of the three cycles following treatment. Acute changes in cycle amplitude were calculated by taking the absolute difference in bioluminescence between the final peak and final trough before treatment (baseline cycle) and the first peak and first trough immediately following treatment (treatment cycle). This was then expressed as the amplitude of the treatment cycle normalized to the baseline cycle.

### Experimental design and statistical analyses.

For Cry1 initiation and pacemaking experiments, the identity of the Cre-expressing AAVs was blinded. Transductions with the Cre-expressing AAVs were randomly allocated to slices of the required genotype in a balanced approach. The final identities of the promoters driving Cre expression were not revealed until after the initial pacemaking and initiation data were acquired and analyzed (see Figs. 2, 3). In all other experiments, no blinding was applied, but where possible slices received paired treatments (vehicle and drug) or were assigned randomly to groups when this was not possible. All data were analyzed in Excel (Microsoft), R (version 3.6.1; R Foundation for Statistical Computing), Rstudio (version 1.2.1335; Rstudio Inc.), Igor Pro 8 (WaveMetrics), and GraphPad Prism 9 (GraphPad). All the statistical tests used are listed in the text and figure legends. All numbers reported in text are mean ± SEM unless otherwise stated.

## Results

### Compromise of astrocytic metabolism reversibly disrupts SCN molecular oscillations

**Figure 1.**
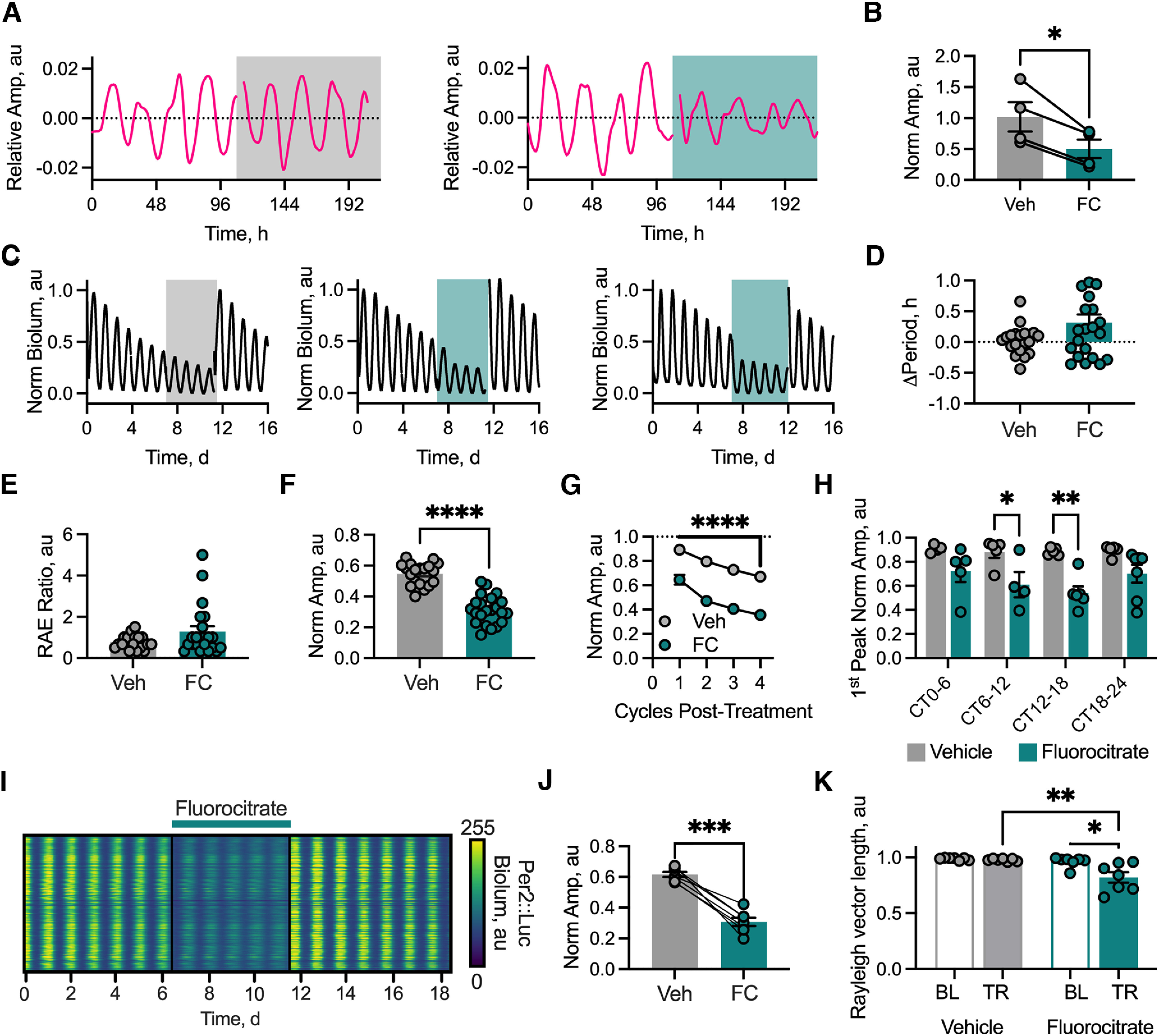
Astrocytic metabolic competence is required for robust, high-amplitude SCN oscillation. ***A***, Example astrocytic calcium rhythms (*GFAP*-cytoGCaMP6f) from slices treated with vehicle (left) or 50 µM fluorocitrate (FC; right) expressed as relative amplitude. Shading indicates the interval of chronic treatment with either vehicle (gray) or 50 µM fluorocitrate (teal). ***B***, Histogram showing paired peak-to-trough amplitude measures of the first cycle of the aggregate calcium rhythm under either vehicle (gray, *N* = 4) or 50 µM fluorocitrate (teal, *N* = 4) treatment normalized to the peak-to-trough amplitude of the cycle preceding treatment. Statistics: paired two-tailed *t* test, *p = 0.018. ***C***, Example normalized PMT traces of aggregate Per2::Luciferase bioluminescent signals showing effects of treating SCN slices with vehicle (left) or 50 µM fluorocitrate (middle, right). Shading indicates the interval of chronic treatment with either vehicle (gray) or 50 µM fluorocitrate (teal). ***D***, Histogram showing period change in hours from baseline, of Per2::Luciferase rhythms induced by treatment with Veh (gray, *N* = 19) or 50 µM FC (teal, *N* = 22). ***E***, Histogram showing the change in RAE ratio (treatment/baseline) of Per2::Luciferase rhythms for Veh-treated (gray, *N* = 19) and 50 µM FC-treated (teal, *N* = 22) slices. ***F***, Histogram showing the normalized amplitude of Per2::luciferase rhythms expressed as the amplitude of the treatment interval normalized to the amplitude of the baseline interval for Veh-treated (gray, *N* = 19) and 50 µM FC-treated (teal, *N* = 22) slices. Statistics: unpaired two-tailed Welch's *t* test, ****p < 0.0001. ***G***, Cycle-to-cycle amplitude of the Per2::Luciferase rhythm normalized to the peak post-treatment for Veh-treated (gray, *N* = 19) and 50 µM FC-treated (teal, *N* = 22) slices. Each point represents the mean ± SEM. Statistics: *post hoc* Sidak's multiple-comparisons test, ****p < 0.0001. ***H***, Histogram showing the amplitude of the first Per2::Luciferase peak of the treatment interval normalized to the last Per2::Luciferase peak of the baseline interval for vehicle-treated (gray) and 50 µM fluorocitrate-treated (teal) slices, separated by phase of treatment. *N* ≥ 4 for each phase. Statistics: Sidak's multiple-comparisons test: *p = 0.031, **p = 0.002. ***I***, False-colored raster plot showing Per2::Luciferase bioluminescence from individual oscillators across the SCN network through time under 50 µM fluorocitrate treatment recorded via CCD camera. Treatment interval is indicated by the teal bar. ***J***, Histogram showing normalized amplitude of aggregate Per2::Luciferase bioluminescence rhythms expressed as the amplitude of the treatment interval normalized to the amplitude of the baseline interval of Veh-treated (gray, *N* = 7) and 50 µM FC-treated (teal, *N* = 7) slices. Paired recordings under vehicle or 50 µM fluorocitrate are indicated by lines connecting individual points. Statistics: paired two-tailed *t* test, ***p = 0.0003. ***K***, Histogram showing Rayleigh vector length determined from network-wide cellular Per2::Luciferase emissions during BL (hollow bars) or treatment (TR; solid bars) intervals. Vehicle treatment (*N* = 7) is indicated by gray coloration, and 50 µM fluorocitrate treatment (*N* = 7) is indicated by teal coloration. Statistics: Sidak's multiple-comparisons test: *p = 0.011, **p = 0.006. In all histogram plots, individual points represent individual slices (with instances where data are paired being joined by lines aside from ***K*** where pairing is not shown for clarity), and histogram bars with error bars represent the mean ± SEM.

In Cry-deficient SCN, astrocytic clocks are, alone, sufficient to initiate rhythmicity (Brancaccio et al., 2019) and to determine circadian period (Brancaccio et al., 2017). It is unclear, however, whether astrocytic metabolic competence is required to maintain the circadian oscillation in an otherwise functional SCN network. To test this, we treated SCN slices with the classical metabolic gliotoxin fluorocitrate. This inhibitor of aconitase (Fonnum et al., 1997) has been shown specifically to disrupt carbon flux through the glial tricarboxylic acid cycle (Swanson and Graham, 1994) and thereby compromise astrocytic function. To confirm this effect of fluorocitrate on SCN astrocytes, we made multiplexed recordings of SCN slices transduced with the specific astrocytic calcium reporter *GFAP*-cytoGCaMP6f, expressed via AAV. This reporter revealed strong circadian rhythms of intracellular calcium ([Ca ^2+^]_i_) in the astrocytes, with a characteristic waveform, as reported previously (Brancaccio et al., 2017; Fig. 1*A*). Over the first 24 h of treatment with 50 µM fluorocitrate, but not vehicle, the amplitude of [Ca^2+^]_i_ rhythms in astrocytes was acutely suppressed (normalized amplitude: vehicle vs fluorocitrate, 1.02 ± 0.24 vs 0.50 ± 0.15; paired two-tailed *t* test: *t*_(3)_ = 4.71, *p* = 0.018; Fig. 1*B*), which persisted with sustained treatment. Treatment of SCN slices with fluorocitrate therefore severely disrupted astrocytic metabolism directly, as reflected by disrupted [Ca^2+^]_i_ rhythms.

Having established the acute effect of fluorocitrate on astrocytic [Ca^2+^]_I_ rhythms within the intact SCN network, we then assessed the effect of this metabolic disruption of astrocytes on the aggregate, network-wide SCN TTFL rhythms. Aggregate bioluminescence rhythms arising from the entire network were recorded in PMTs from Per2::Luciferase SCN before, during, and after 5 d of treatment with either vehicle or 50 µM fluorocitrate (Fig. 1*C*). Fluorocitrate treatment did not alter the overall period (median (Q1,Q3) Δperiod from baseline: vehicle vs fluorocitrate, 0.03 (−0.14,0.13) vs 0.20 (−0.25,0.78) h; Mann–Whitney test, U = 158.5, *p* = 0.19; Fig. 1*D*) or the precision of the oscillation, as determined by the relative amplitude error (RAE; RAE ratio treatment/baseline: vehicle vs fluorocitrate, 0.78 ± 0.08 vs 1.28 ± 0.26 a.u., unpaired two-tailed Welch's *t* test: *t*_(24.51)_ = 1.85, *p* = 0.08; Fig. 1*E*). It did, however, significantly reduce the amplitude of the aggregate Per2::Luciferase rhythm by ∼50%, compared with vehicle-treated slices (Fig. 1*C*,*F*; normalized amplitude: vehicle vs fluorocitrate 0.55 ± 0.02 vs 0.31 ± 0.02 a.u.; unpaired two-tailed Welch's *t* test: *t*_(38.72)_ = 9.08, *p* < 0.0001). Notwithstanding the gradual reduction in amplitude arising from luciferin depletion from the medium (two-way ANOVA time effect: *F*_(3,112)_ = 80.36, *p* < 0.0001), the effect of fluorocitrate was immediate and was sustained (two-way ANOVA treatment effect: *F*_(1,39)_ = 127.4, *p* < 0.0001; Fig. 1*G*) until its removal by medium change. Consequently, there was no significant interaction between time and treatment (two-way ANOVA time-by-treatment interaction, *F*_(3,112)_ = 2.049, *p* = 0.1). Upon medium change, the normalized amplitude under treatment with vehicle reversed from 55 ± 7% to 97 ± 2% of the baseline amplitude, while the reduction in normalized amplitude observed under fluorocitrate treatment was reversed from 31 ± 9% to 80 ± 6% of the normalized amplitude. Furthermore, the immediate suppressive effect of fluorocitrate on network-wide TTFL rhythms was phase dependent, insofar as the reduced amplitude of the first peak following treatment was greatest when SCNs were treated during the late circadian day and early circadian night [circadian time 6 (CT6) to CT12 and CT12 to CT18; Fig. 1*C*,*H*], phases that correspond to the time of increasing astrocytic activity (Brancaccio et al., 2017). In SCNs where treatment missed this window of sensitivity, peak amplitude was not suppressed until the second cycle. These data indicate that the robustness of circadian oscillation in the SCN is dependent on astrocytic metabolism, and that compromise of astrocytic metabolism can reversibly suppress the TTFL, likely by reducing the network reinforcement of circadian amplitude. We then investigated whether the disruption caused by fluorocitrate manifested as changes in network-level synchrony by imaging Per2::Luciferase bioluminescence via CCD camera. As with the ensemble signal from PMT recordings (Fig. 1*C*), treatment with fluorocitrate reduced the amplitude of TTFL rhythms in individual oscillators across the network (Fig. 1*I*,*J*; normalized amplitude vehicle vs fluorocitrate, 0.61 ± 0.02 vs 0.31 ± 0.03 a.u.; paired two-tailed *t* test: *t*_(6)_ = 9.38, *p* < 0.0001). This reduction in amplitude was associated with a significant reduction in synchrony under treatment with fluorocitrate, as measured by Rayleigh vector length (two-way repeated-measures ANOVA, treatment effect: *F*_(1,6)_ = 10.72, *p* = 0.017), such that SCNs treated with fluorocitrate were less synchronized than before treatment (two-way repeated-measures ANOVA, interval effect: *F*_(1,6)_ = 15.75, *p* = 0.007) and less than vehicle-treated slices (two-way repeated-measures ANOVA, treatment-by-interval interaction: *F*_(1,6)_ = 12.3, *p* = 0.013; Fig. 1*K*). This reduction in synchrony is consistent with weaker cycle-on-cycle reinforcement of cellular rhythms across the network caused by the reduced amplitude of the SCN TTFL, itself an effect of compromised astrocytic metabolism. Metabolically compromised astrocytes therefore lead to a weaker clock network.

### Initiation of *de novo* rhythmicity in Cry-null SCN by astrocytes

**Figure 2.**
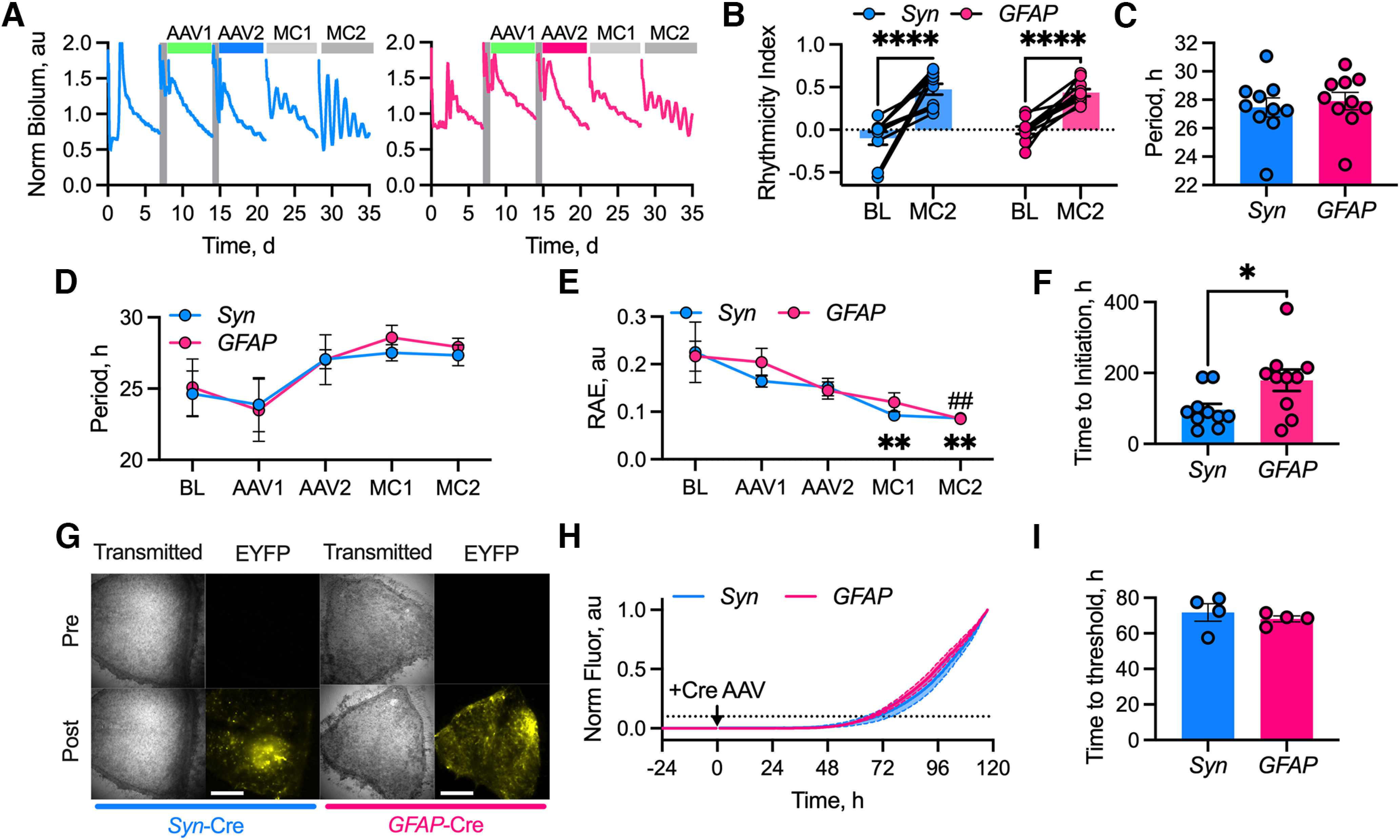
Neurons and astrocytes initiate *de novo* rhythmicity in the SCN at different rates. ***A***, Example PMT traces showing progression of the experiment for Cry-null SCN transduced with *Syn*-mCherry::Cre (left, blue) and *GFAP*-mCherry::Cre (right, pink). Medium changes preceding AAV transduction are shown by shaded gray areas. AAV transductions and medium changes following the serial transduction are indicated by colored bars (from left to right): AAV1 (*pCry1*-DIO.Cry1::EGFP, green), AAV2 (*Syn-*mCherry::Cre, blue or *GFAP*-mCherry::Cre, pink), and MC1 and MC2 (gray). ***B***, Histogram showing rhythmicity index for the noninitiated BL interval and the initiated slices in the final interval of the experiment (MC2) for *Syn*-mCherry::Cre (*Syn*, blue, *N* = 10) and *GFAP*-mCherry::Cre (*GFAP*, pink, *N* = 10). Statistics: Sidak's multiple-comparisons test, ****p < 0.0001. ***C***, Histograms showing final initiated periods for neuron-initiated (*Syn*, blue, *N* = 10) and astrocyte-initiated (*GFAP*, pink, *N* = 10) SCN slices. ***D***, Summary FFT-determined period data, showing that SCN slices transduced with *Syn*-mCherry::Cre (*Syn*, blue, *N* = 10) or *GFAP*-mCherry::Cre (*GFAP*, pink, *N* = 10). Intervals plotted along the *x*-axis correspond to the experimental intervals. ***E***, Summary FFT-determined RAE measures, showing *Syn*-mCherry::Cre (*Syn*, blue, *N* = 10) and *GFAP*-mCherry::Cre (*GFAP*, pink, *N* = 10). Intervals plotted along the *x*-axis correspond to the experimental intervals. Statistics: Dunnett's multiple-comparisons test: neurons, **p < 0.01 versus BL; astrocytes, ##*p* = 0.004 versus BL. ***F***, Histogram showing the time in hours post-transduction with the AAV-Cre to the initiation of rhythmicity as determined by wavelet-based analysis for *Syn*-mCherry::Cre (*Syn*, blue, *N* = 10) and *GFAP*-mCherry::Cre (*GFAP*, pink, *N* = 10). Statistics: unpaired Welch's *t* test, *p* = 0.03. ***G***, Example images from SCN slices transduced with *EF1a*.DIO.EYFP pretransduction or 5 d post-transduction with either *Syn*-mCherry::Cre (left, blue) or *GFAP*-mCherry::Cre (right, pink). Scale bar, 250 µm. ***H***, Aggregate time course of normalized fluorescence intensity over 5 d post-AAV-Cre transductions for *Syn*-mCherry::Cre (*Syn*, blue, *N* = 4) and *GFAP*-mCherry::Cre (*GFAP*, pink, *N* = 4). Dashed line indicates a 10% increase in intensity threshold. ***I***, Histogram showing the time post-transduction for the normalized fluorescence intensity to pass the 10% threshold for *Syn*-mCherry::Cre (*Syn*, blue, *N* = 4) and *GFAP*-mCherry::Cre (*GFAP*, pink, *N* = 4). In all plots, individual points indicate individual SCN with instances where data are paired being connected by lines. In ***D*** and ***E***, the points represent the mean ± SEM. In all plots, lines with shading enclosed by dashed lines and histogram bars with error bars represent the mean ± SEM.

Having established that astrocytic metabolic competence is required for proper SCN function, even when both astrocytes and neurons have functional TTFLs, we sought to test the relative contributions of neurons and astrocytes to the *de novo* initiation of rhythmicity in the SCN. We expressed Cre-conditional Cry1 under the control of the minimal Cry1 promoter (*pCry1*-DIO.Cry1::EGFP) delivered via AAVs into Cry1,2-null (Cry-null), Per2::Luciferase-positive SCN explants. To express Cry1 specifically in these two cell types, Cre-recombinase was expressed under the control of either the human synapsin promoter (*Syn*; *Syn*-mCherry::Cre) or the short GFAP promoter (*GFAP*; *GFAP*-mCherry::Cre) to target neurons or astrocytes, respectively. Cry-deficient SCN slices were arrhythmic during the baseline interval, up until cell type-specific Cre released conditional Cry1::EGFP expression (Fig. 2*A*). Rhythmicity was initiated following the addition of Cre to either population, stabilizing over the next 20 d, and evidenced by an increase in the rhythmicity index (autocorrelation at 28 h; two-way ANOVA interval effect: *F*_(1,18)_ = 63.56, *p* < 0.0001). By this measure, there was no difference in quality between the rhythms generated by the two cell types (two-way ANOVA cell-type effect: *F*_(1,18)_ = 0.28, *p* = 0.60; Fig. 2*B*). Furthermore, the rhythmicity initiated by both populations stabilized with an identical period of ∼27 h, regardless of the cell type targeted (neuron-initiated vs astrocyte-initiated, 27.5 ± 0.7 vs 27.9 ± 0.6 h; unpaired two-tailed *t* test *t*_(18)_ = 0.5, *p* = 0.6; Fig. 2*C*). Having established that both populations can initiate rhythmicity, we then assessed this initiation in intervals of 7 d, corresponding to the experimental periods covering BL, post-Cry AAV transduction (AAV1), post-Cre AAV transduction (AAV2), and two serial MCs (MC1 and MC2; Fig. 2*A*). FFT-based analysis of PMT recordings were assigned nominal periods ranging between 10 and 40 h in the windows before Cre-AAV addition, which then converged (two-way ANOVA time effect: *F*_(4,65)_ = 3.74, *p* = 0.009) at ∼27 h following the targeting of Cre to either cell type (two-way ANOVA cell type effect: *F*_(1,18)_ = 0.09, *p* = 0.76; Fig. 2*D*).

As the FFT-based analysis inevitably assigns nominal periods to the arrhythmic portion of the recording, it is not possible to quantify whether there is a difference in initiation kinetics between the two cell populations based solely on the FFT-reported period. We therefore examined the identified rhythms, using the inverse metric of quality, RAE (Fig. 2*E*). As with period, RAE displayed a large range during the arrhythmic intervals before Cre was added. Following addition of the Cre-AAV, however, rhythms became better organized in both cases and the RAE values converged (two-way ANOVA time effect: *F*_(4,65)_ = 8.19, *p* < 0.0001) to the same level (neurons, 0.09 ± 0.01; astrocytes, 0.09 ± 0.01; two-way ANOVA cell type effect: *F*_(1,18)_ = 0.27, *p* = 0.61). Furthermore, *post hoc* multiple-comparisons test to the baseline interval revealed that the neuronally initiated slices reached their final RAE measurement sooner than did the astrocyte-initiated slices [neurons, 7–14 d post-transduction (dpt; MC1); astrocytes, 14–21 dpt (MC2); Fig. 2*E*]. To assess the difference in the kinetics of *de novo* rhythmicity more formally, we applied a wavelet-based analysis (Price et al., 2008) to determine the time at which the algorithm detected rhythms with an instantaneous period within the circadian range of 25–29 h. As determined by these criteria, rhythmicity was initiated in all slices, but the rate differed between the two cell types. The point at which the instantaneous period crossed into the 25–29 h threshold showed that neurons initiated robust rhythmicity sooner than did astrocytes (neurons vs astrocytes, 96.6 ± 16.5 vs 179.5 ± 30.1 h; unpaired Welch's two-tailed *t* test: *t*_(13.96)_ = 2.41, *p* = 0.03; Fig. 2*F*).

This difference in kinetics could arise from the cell type targeted or from the relative efficiency of the cell-specific promoters driving the Cre-recombinase. To assess this, we transduced SCN slices with an AAV to Cre-conditionally express EYFP (*EF1a*-DIO.EYFP) and recorded a baseline. In the absence of Cre-recombinase, there was no detectable expression of EYFP (Fig. 2*G*,*H*). Once either *Syn*- or *GFAP*-driven Cre-expressing AAVs were added, however, EYFP fluorescence emerged and revealed cell type-specific morphologies (Fig. 2*G*). Moreover, it increased over the following 5 d at a comparable rate in both populations (Fig. 2*H*). To quantify the kinetics of this rise, we determined the time post-transduction for the normalized fluorescence signal to pass a threshold of a 10% increase in signal. There was no significant difference in the time taken to reach this point between the two cell type-specific promoters (*Syn* vs *GFAP*, 71.75 ± 4.97 vs 68.13 ± 1.68 h; unpaired Welch's two-tailed *t* test: *t*_(3.672)_ = 0.69, *p* = 0.53; Fig. 2*H*,*I*), indicating that differences in the rate of initiation of rhythmicity by either neurons or astrocytes cannot be attributed to differential expression of Cre-recombinase from the *Syn* or *GFAP* promoters, respectively. Thus, while both neurons and astrocytes can initiate *de novo* rhythmicity with the same period and quality in an otherwise clockless SCN, astrocytes take much longer than do neurons to impose their cell-autonomous TTFL timekeeping across the full circuit.

### Bidirectional control of SCN period by astrocytes

**Figure 3.**
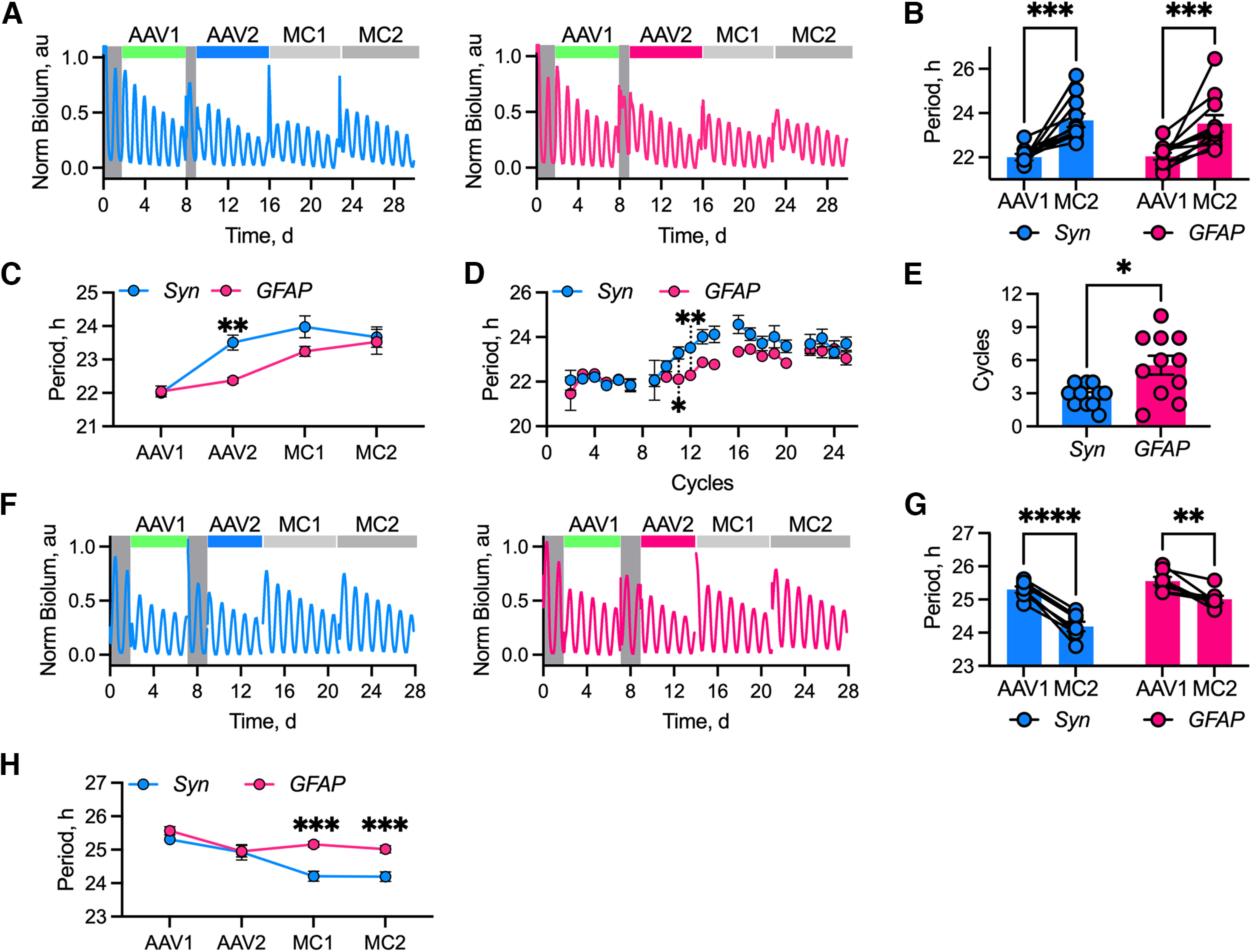
Neurons are more potent SCN pacemakers than astrocytes. ***A***, Example PMT traces showing progression of the experiment for Cry1-null SCN transduced with *Syn*-mCherry::Cre (left, blue) and *GFAP*-mCherry::Cre (right, pink). Medium changes preceding AAV transduction are shown by shaded gray areas. AAV transductions and medium changes following the serial transduction are indicated by colored bars (from left to right): AAV1 (*pCry1*-DIO.CRY1::EGFP, green), AAV2 (*Syn-*mCherry::Cre, blue or *GFAP*-mCherry::Cre, pink), and MC1 and MC2 (medium changes, gray). ***B***, Histogram showing paired first interval (AAV1) and final post-Cre interval (MC2) aggregate periods for targeted neurons (*Syn*, blue, *N* = 11) and astrocytes (*GFAP*, pink, *N* = 11). Statistics: Sidak's multiple-comparisons test, ***p < 0.001. ***C***, Summary period data for SCN slices transduced with *Syn*-mCherry::Cre (*Syn*, blue, *N* = 11) or *GFAP*-mCherry::Cre (*GFAP*, pink, *N* = 11). Intervals plotted along the *x*-axis correspond to the experimental intervals. Statistics: Sidak's multiple-comparisons test, **p = 0.002 *Syn* versus *GFAP*. ***D***, Summary peak-to-peak period data, showing SCN slices transduced with *Syn*-mCherry::Cre (*Syn,* blue, *N* = 11) or *GFAP*-mCherry::Cre (*GFAP*, pink, *N* = 11). Statistics: Sidak's multiple-comparisons test, *p = 0.049, **p = 0.001 *Syn* versus *GFAP*. ***E***, Histogram showing the number of cycles post-transduction with either *Syn*-mCherry::Cre (*Syn*, blue, *N* = 11) or *GFAP*-mCherry::Cre (*GFAP,* pink, *N* = 11) for slices to achieve the half-maximal period change. Statistics: unpaired Welch's *t* test, *p = 0.011. ***F***, Example PMT traces showing the progression of the experiment for Cry2-null SCN transduced with *Syn*-mCherry::Cre (left, blue) and *GFAP*-mCherry::Cre (right, pink). Medium changes preceding AAV transduction are shown by shaded gray areas. AAV transductions and medium changes following the serial transduction are indicated by colored bars (from left to right): AAV1 (*pCry2*-DIO.CRY2::EGFP, green), AAV2 (*Syn-*mCherry::Cre, blue; or *GFAP*-mCherry::Cre, pink), and MC1 and MC2 (gray). ***G***, Histogram showing paired BL and final post-Cre (MC2) aggregate periods for targeted neurons (*Syn*, blue, *N* = 7) and astrocytes (*GFAP*, pink, *N* = 7). Statistics: Sidak's multiple-comparisons test, **p = 0.002, ****p < 0.0001. ***H***, Summary period data for SCN slices transduced with *Syn*-mCherry::Cre (*Syn*, blue, *N* = 7) or *GFAP*-mCherry::Cre (*GFAP*, pink, *N* = 7). Intervals plotted along the *x*-axis correspond to the experimental intervals. Statistics: Sidak's multiple-comparisons test, ***p < 0.001 *Syn* versus *GFAP*. In all histogram plots, individual points represent individual slices (with instances where data are paired being joined by lines), and histogram bars with error bars represent the mean ± SEM. In ***C***, ***D***, and ***H***, points are the mean ± SEM.

The network of Cry1,2-null circadian-incompetent neurons may provide a permissive context for the imposition of astrocytic cell-autonomous timekeeping across the SCN. A potentially more demanding test of astrocytic influence is the ability to impose their cell-autonomous properties on an otherwise circadian-competent SCN. We therefore applied the same dual-transduction approach to express either Cry1 or Cry2 in an attempt to lengthen or shorten the period of rhythmic short-period Cry1-null or long-period Cry2-null SCN, respectively. How effectively can astrocytes act as pacemakers and determine the period of an ongoing stable oscillation? In the absence of any Cre expression, Cry1-null SCN slices transduced with *pCry1*-DIO.Cry1::EGFP exhibited a short (∼22 h) period (Fig. 3*A*,*B*). Cell-selective expression of Cre-recombinase lengthened the period of both treatment groups over the following 20 d, to ultimately stabilize at ∼23.5 h (two-way ANOVA interval effect: *F*_(1,20)_ = 39.09, *p* < 0.0001; Fig. 3*B*), and with no difference between SCNs in which either of the two cell types was targeted (two-way ANOVA cell-type effect: *F*_(1,20)_ = 0.03, *p* = 0.86; Fig. 3*B*,*C*). Coarse, longitudinal assessment of FFT-assessed period over successive time windows confirmed that the period lengthened progressively (two-way ANOVA interval effect: *F*_(3,60)_ = 33.46, *p* < 0.0001), and the final period was not significantly different between the two targeted cell populations (two-way ANOVA cell type effect: *F*_(1,20)_ = 3.64, *p* = 0.07). However, despite the fact that both populations could equally lengthen the period, explants in which neurons were targeted reached their stable final period at an earlier experimental interval than did SCNs in which the period was lengthened by the astrocytes (two-way ANOVA cell type/interval interaction: *F*_(3,60)_ = 4.35, *p* = 0.008; Fig. 3*C*). To assess more precisely how the period changed, we calculated the peak-to-peak intervals for neuron- or astrocyte-targeted SCNs (Fig. 3*D*). Again, both cell types produced equivalent period changes (repeated-measures two-way ANOVA cell type effect: *F*_(1,20)_ = 3.43, *p* = 0.08), with significant lengthening following the addition of the AAV-Cre (repeated-measures two-way ANOVA time effect: *F*_(4.93,93.13)_ = 17.17, *p* < 0.0001; Fig. 3*D*). Consistent with the previous observation, however, there was a significant difference in the temporal dynamics required to achieve this period change between the two cell-types: a neuron-targeted SCN lengthened period more rapidly than astrocyte-targeted SCN (repeated-measures two-way ANOVA cell type-by-time interaction: *F*_(21,397)_ = 2.25, *p* = 0.001; Fig. 3*D*). Having established that neurons determine period much more rapidly than do astrocytes, we refined this observation by determining the number of cycles post-Cre transduction at which the peak-to-peak period lengthened beyond the point of the half-maximal period change (Fig. 3*E*). Neuronally targeted SCNs reached this point in about half the number of cycles that it took for astrocytically targeted SCNs (neurons vs astrocytes, 2.8 ± 0.3 vs 5.3 ± 0.6 cycles; unpaired Welch's two-tailed *t* test: *t*_(12.35)_ = 3.01, *p* = 0.011; Fig. 3*E*). Notwithstanding the more demanding context of a functional rather than circadian-incompetent SCN circuit, the relative rate of the pacemaking exerted by the cell-autonomous astrocytic clock was again approximately half as fast as that of the neuronal clock. TTFL-encoded timing cues from astrocytes can slow the period of the whole SCN, but take longer than do neuronally derived cues. Perhaps the cues have different intrinsic potencies, and/or astrocytes are more readily controlled (slowed) by neurons, than vice versa.

From a conceptual perspective, it might be expected that slowing down circadian timekeeping, which could be achieved by inhibiting a single rate-limiting process, may be easier than accelerating it, which would require all processes to be regulated simultaneously. Indeed, most tests of neuronal pacemaking have relied on lengthening their cell-autonomous TTFL (Lee et al., 2015; Smyllie et al., 2016; Brancaccio et al., 2017, 2019; van der Vinne et al., 2018; Patton et al., 2020; Hamnett et al., 2021). As a final comparison of neuronal and astrocytic pacemaking, we therefore exploited genetic complementation of Cry2, in either neurons or astrocytes, to shorten the period of Cry2-null SCN. This would not only test the bidirectional effect of cell-autonomous neuronal and astrocytic clocks, but also provide a potentially more resistant context for this comparison. Bioluminescence rhythms of Cry2-null SCNs exhibited characteristically long periods (∼26 h) in the presence of AAV *pCry2*-DIO.CRY2::EGFP AAV (Fig. 3*F*). On addition of neuronally specific AAV-Cre, the period progressively shortened over the subsequent 20 d (Fig. 3*F*,*G*) to 24.2 ± 0.15 h. Addition of the astrocyte-specific AAV-Cre also caused the period to shorten (two-way ANOVA interval effect: *F*_(1,12)_ = 85.25, *p* < 0.0001), but the final stable period (25.01 ± 0.15 h) was significantly longer than the neuronally targeted SCN (Fig. 3*G*; two-way ANOVA cell type effect: *F*_(1,12)_ = 14.4, *p* = 0.003; cell type-by-interval interaction: *F*_(1,12)_ = 9.70, *p* = 0.009). FFT-based analysis across the experiment confirmed that both populations were able to shorten period progressively (two-way ANOVA interval effect: *F*_(3,36)_ = 19.41, *p* < 0.0001), but were not equally effective (two-way ANOVA cell type effect: *F*_(1,12)_ = 12.96, *p* = 0.004; cell type-by-interval interaction: *F*_(3,36)_ = 6.72, *p* = 0.001; Fig. 3*H*). These experiments reveal, first, that the cell-autonomous clock of either neurons or astrocytes can shorten, as well as lengthen, the ensemble period on the SCN and, second, that astrocytes are much less potent than neurons in this regard. In the case of lengthening period, both cell types are ultimately equally effective, albeit neurons are effective sooner. With potentially more demanding period shortening, although both populations can shorten the period to some extent, astrocytes are much weaker pacemakers than are neurons, such that the emergent ensemble period more closely matches that of the period of the surrounding untargeted neurons.

### Chemogenetic manipulation of astrocytic cellular activity

**Figure 4.**
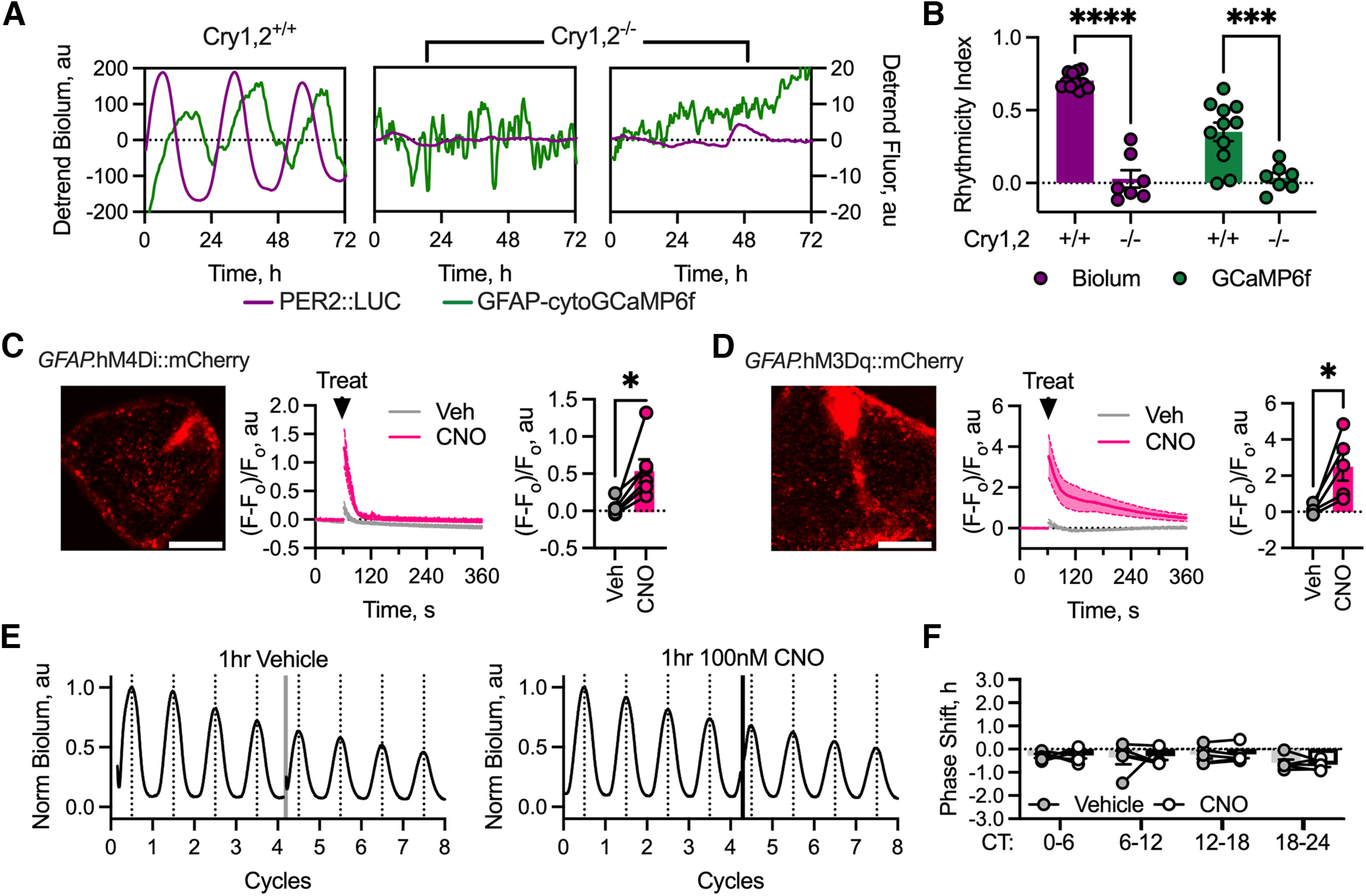
Acute activation of DREADDs in astrocytes triggers astrocytic calcium responses. ***A***, Example traces showing Per2::Luciferase bioluminescence (purple) and *GFAP*-cytoGCaMP6f fluorescence (green) from a wild-type (Cry1,2^+/+^, left) and two Cry-null (Cry1,2^−/−^, middle and right) SCN. ***B***, Histogram showing the rhythmicity index for PER2::Luciferase bioluminescence (purple) and *GFAP*-cytoGCaMP6f (green) from wild-type (Cry1,2^+/+^, *N* = 11) or Cry-null (Cry1,2^−/−^, *N* = 7) SCN. Statistics: Sidak's multiple-comparisons test, ***p = 0.0002, ****p < 0.0001. ***C***, Left, Example false-colored image showing astrocytic hM4Di::mCherry expression (*GFAP*-hM4Di::mCherry). Middle, Aggregate GCaMP6f fluorescence change from SCN slices expressing astrocytic hM4Di following either Veh (gray, *N* = 6) or 100 nm CNO (pink, *N* = 6) treatment expressed as (*F* – *F*_0_)/*F*_0_. Scale bar, 250 µm. Right, Histogram showing the mean fluorescence change over the first 30 s of aggregate GCaMP fluorescence following either Veh (gray, *N* = 6) or 100 nm CNO (pink, *N* = 6) treatment. Statistics: paired two-tailed *t* test, *p = 0.019. ***D***, Left, Example false-colored image showing astrocytic hM3Dq::mCherry expression (*GFAP*-hM3Dq::mCherry). Scale bar, 250 µm. Middle, aggregate GCaMP6f fluorescence change from SCN slices expressing astrocytic hM3Dq following either Veh (gray, *N* = 4) or 100 nM CNO (pink, right, *N* = 4) treatment expressed as (*F* – *F*_0_)/*F*_0_. Right, histogram showing the mean fluorescence change of aggregate GCaMP fluorescence over the first 30 s following either Veh (gray, *N* = 4) or CNO (pink, *N* = 4) treatment. Statistics: paired two-tailed *t* test, *p = 0.03. ***E***, Representative PMT traces showing acute vehicle (left) and 100 nm CNO (right) treatment during the CT0 to CT6 time window. The treatment interval is shown as a vertical line and colored according to treatment: vehicle (gray) or CNO (black). ***F***, Summary phase shift data arranged by phase window of treatment showing paired recordings at that phase window for untransduced SCN explants treated with vehicle (gray, *N* = 5 at each phase) or 100 nm CNO (black/white, *N* = 5 at each phase). In all plots, lines with shading encased by dashed lines represent the mean ± SEM, individual points represent independent slices, and histogram bars with error bars represent the mean ± SEM. In ***C***, ***D*,** and ***F***, joined points represent paired measures.

Disrupting astrocytic metabolism, initiating rhythmicity *de novo* and changing the ensemble period are chronic manipulations of the ongoing SCN oscillation, but to what extent can acute changes to the cellular activity of neurons and astrocytes alter the phase of SCN timekeeping? We therefore expressed DREADDs to manipulate neurons or astrocytes chemogenetically via Gi- or Gq-coupled receptors. Chemogenetic targeting of SCN neurons has been reported previously (Brancaccio et al., 2013), but to determine its efficacy in SCN astrocytes, we expressed GCaMP6f under the control of the GFAP promoter to monitor astrocytic [Ca^2+^]_i_. Using Per2::Luciferase as a circadian reference point, we reconfirmed circadian oscillations of [Ca^2+^]_i_ in astrocytes (Fig. 4*A*) with the appropriate period (Per2::Luciferase, 24.4 ± 0.1 h; GCaMP6f, 24.3 ± 0.1 h, *n* = 11; paired two-tailed *t* test: *t*_(10)_ = 0.93, *p* = 0.37) and a nocturnal peak (CT17.6 ± 0.3 h, *n* = 11). In Cry-null SCN, the rhythm of Per2::Luciferase bioluminescence was lost, such that its rhythmicity index (autocorrelation at 24 h) was significantly reduced (wild-type vs Cry-null: 0.70 ± 0.02 vs 0.03 ± 0.06 a.u.; unpaired Welch's two-tailed *t* test: *t*_(6.892)_ = 10.82, *p* < 0.0001). Equally, the circadian cycle of [Ca^2+^]_i_ in astrocytes was also absent in Cry-null SCN, with a reduced rhythmicity index (wild-type vs Cry-null, 0.35 ± 0.06 vs 0.04 ± 0.04 a.u., unpaired Welch's two-tailed *t* test: *t*_(14.61)_ = 4.34, *p* = 0.0006; Fig. 4*B*). The astrocytic [Ca^2+^]_i_ rhythm is therefore dependent on the SCN TTFL. We then used Cry-null SCNs to assess the efficacy of acute chemogenetic manipulation of astrocytic activity, reported as [Ca^2+^]_i_, in the absence of any confound from circadian oscillations. The Gi-coupled DREADD hM4Di, delivered to SCN astrocytes via AAV (*GFAP*-hM4Di::mCherry), exhibited appropriate astrocytic distribution (Fig. 4*C*), in close register to astrocytic GCaMP6f. On dropwise addition of vehicle, astrocytic [Ca^2+^]_i_ fluctuated transiently, but rapidly returned to baseline levels (Fig. 4*C*). In contrast, addition of 100 nM CNO rapidly elevated aggregate astrocytic [Ca^2+^]_i_ and activated cells across the SCN, an effect that persisted for ∼30 s before a return to baseline levels (Fig. 4*C*). Consequently, the mean [Ca^2+^]_i_ level over the first 30 s following acute treatment exhibited a significant elevation, compared with vehicle (Veh vs CNO, 0.04 ± 0.04 vs 0.57 ± 0.16 a.u.; paired two-tailed *t* test: *t*_(5)_ = 3.43, *p* = 0.019; Fig. 4*C*). We next tested the ability of the Gq-coupled DREADD hM3Dq (*GFAP*-hM3Dq::mCherry) to manipulate astrocytic [Ca^2+^]_i_. Again, this revealed an appropriate astrocytic distribution of mCherry fluorescence (Fig. 4*D*), and dropwise addition of vehicle caused a transient fluctuation of astrocytic [Ca^2+^]_i_ before it returned to baseline levels (Fig. 4*D*). In contrast, the addition of 100 nm CNO caused an immediate and sustained elevation of astrocytic [Ca^2+^]_i_ (Fig. 4*D*). Again, the mean CNO-induced elevation of astrocytic [Ca^2+^]_i_ over the first 30 s of treatment was significantly elevated relative to vehicle (Veh vs CNO, 0.11 ± 0.11 vs 2.52 ± 0.78 a.u.; paired two-tailed *t* test: *t*_(4)_ = 3.33, *p* = 0.03; Fig. 4*D*). Thus, Gi- and Gq-coupled DREADDs are functional in SCN astrocytes, and although they both trigger elevations of [Ca^2+^]_i_, these responses are qualitatively different, indicating that Gi- and Gq-coupled DREADDs activate distinct intracellular pathways in astrocytes.

### Differential chemogenetic determination of SCN ensemble phase by neurons and astrocytes

We then used chemogenetic manipulation of neurons or astrocytes to compare their relative contributions to the determination of SCN ensemble phase. Because of reported off-target effects of CNO treatment caused by conversion of CNO to clozapine in murine tissues (Manvich et al., 2018), we assessed any nonspecific effects of CNO treatment using untransduced Per2::Luciferase SCN slices treated across circadian time. After baseline recording of circadian bioluminescence, SCNs were treated with vehicle or CNO (100 nM) in culture medium for 1 h by transfer to a separate culture dish. Following serial washout, the slices were returned to their original culture dish and luciferin-containing medium, and recording was continued (Fig. 4*E*). These experiments revealed that there is no effect on ensemble phase when SCN explants are treated with vehicle or CNO in this way (repeated-measures two-way ANOVA treatment effect: *F*_(1,16)_ = 0.0004, *p* = 0.98); nor was there any dependence on phase of treatment (repeated-measures two-way ANOVA phase effect: *F*_(3,16)_ = 2.10, *p* = 0.14; treatment-by-time interaction: *F*_(3,16)_ = 0.07, *p* = 0.98; Fig. 4*F*).

**Figure 5.**
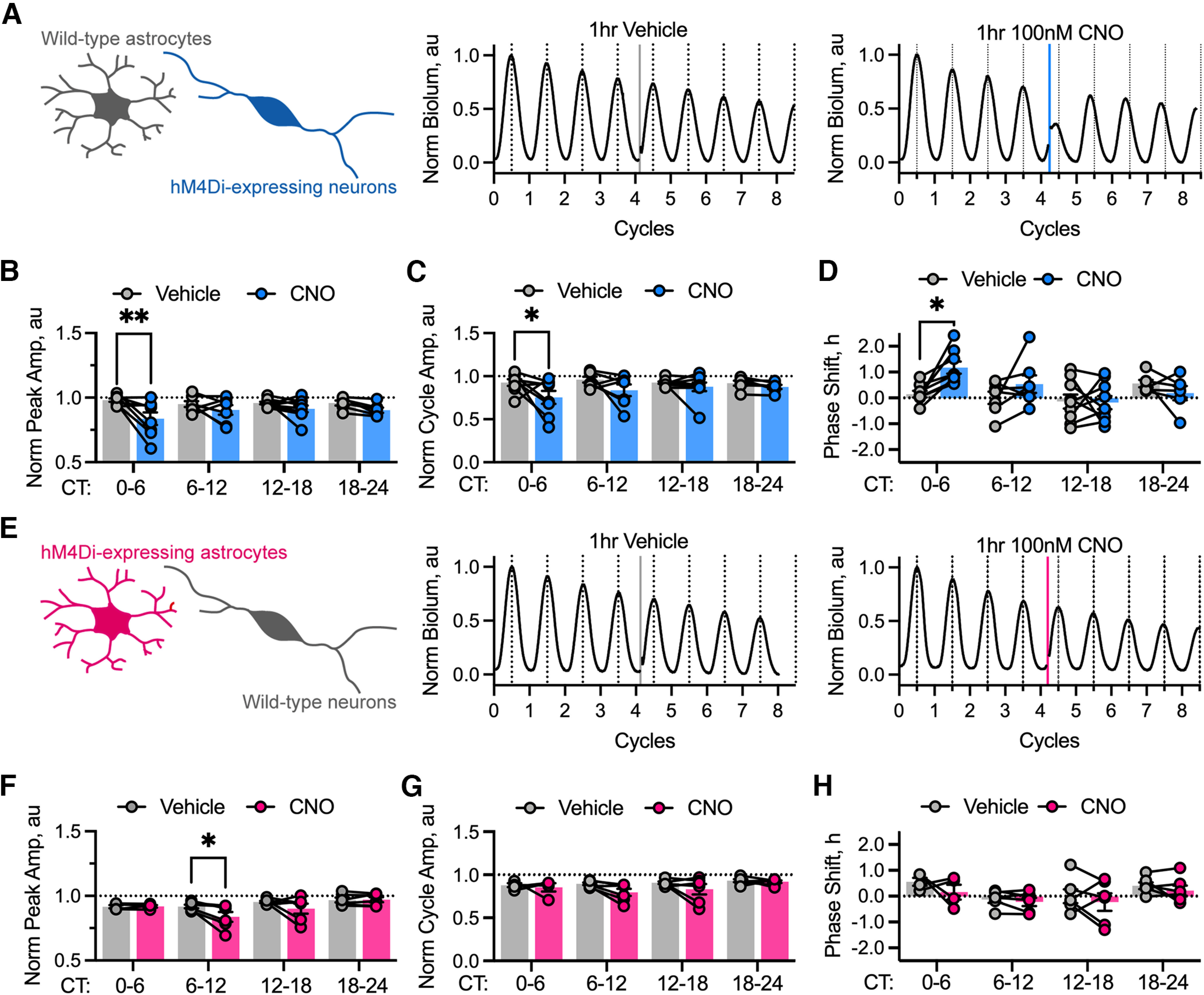
Chemogenetic activation of a Gi-coupled pathway in neurons, but not astrocytes, resets SCN ensemble phase. ***A***, Schematic showing cell type-specific targeting of Gi-coupled DREADDs to neurons (*Syn*-hM4Di::mCherry), but not astrocytes in the intact SCN network (left) alongside representative PMT traces showing acute vehicle (middle) and 100 nM CNO (right) treatment during the CT0 to CT6 time window. The treatment interval is shown as a vertical line and colored according to treatment: vehicle (gray) or CNO (blue). ***B***, Summary peak amplitude data arranged by phase window of treatment showing paired recordings at that window for SCN with neuronally expressed Gi-coupled DREADDs treated with vehicle (gray, *N* ≥ 6 at each phase) or 100 nM CNO (blue, N ≥ 6 at each phase). Statistics: Sidak's multiple-comparisons test, **p = 0.0027. ***C***, Summary cycle amplitude data arranged by phase window of treatment showing paired recordings at that window for SCN with neuronally expressed Gi-coupled DREADDs treated with vehicle (gray, *N* ≥ 6 at each phase) or 100 nm CNO (blue, *N* ≥ 6 at each phase). Statistics: Sidak's multiple-comparisons test, *p = 0.0421. ***D***, Summary phase-shift data arranged by phase window of treatment showing paired recordings at that phase window for SCN with neuronally expressed Gi-coupled DREADDs treated with vehicle (gray, *N* ≥ 6 at each phase) or 100 nM CNO (blue, *N* ≥ 6 at each phase). Statistics: Sidak's multiple-comparisons test, *p = 0.019. ***E***, Schematic showing cell type-specific targeting of Gi-coupled DREADDs to astrocytes (*GFAP*-hM4Di::mCherry), but not neurons in the intact SCN network (left) alongside representative PMT traces showing acute vehicle (middle) and 100 nM CNO (right) treatment during the CT0 to CT6 time window. The treatment interval is shown as a vertical line and colored according to treatment: vehicle (gray) or CNO (pink). ***F***, Summary peak amplitude data arranged by phase window of treatment showing paired recordings at that window for SCN with astrocytically expressed Gi-coupled DREADDs treated with vehicle (gray, *N* ≥ 4 at each phase) or 100 nm CNO (pink, *N* ≥ 4 at each phase). Statistics: Sidak's multiple-comparisons test, *p = 0.0401. ***G***, Summary cycle amplitude data arranged by phase window of treatment showing paired recordings at that window for SCN with astrocytically expressed Gi-coupled DREADDs treated with vehicle (gray, *N* ≥ 4 at each phase) or 100 nM CNO (pink, *N* ≥ 4 at each phase). ***H***, Summary phase-shift data arranged by phase window of treatment showing paired recordings at that phase window for SCN with astrocytically expressed Gi-coupled DREADDs treated with vehicle (gray, *N* ≥ 4 at each phase) or 100 nm CNO (pink, *N* ≥ 4 at each phase). In all histogram plots, individual points joined by lines represent individual slices with paired treatment and histogram bars with error bars represent mean ± SEM.

Having established chemogenetic manipulation of astrocytes and neurons as viable tools for studying phase determination in the SCN, we first expressed the Gi-coupled DREADD (hM4Di) under the *Syn* promoter in neurons of Per2::Luciferase SCN slices. Neuronal Gi-activation had both acute and sustained effects in slices treated across circadian time (Fig. 5*A*). The height of the first peak following treatment was strongly reduced by CNO treatment (Fig. 5*B*; repeated-measures two-way ANOVA treatment effect: *F*_(1,8)_ = 11.37, *p* = 0.0098), but this did not appear to be associated with the phase at which treatment was given (repeated-measures two-way ANOVA phase effect: *F*_(3,24)_ = 0.38, *p* = 0.77) or with an interaction (repeated-measures two-way ANOVA, treatment-by-phase interaction: *F*_(3,12)_ = 2.01, *p* = 0.17). However, looking at how this acute suppression of the peak manifested across recordings, *post hoc* testing revealed a specific effect when slices were treated in early circadian day, CT0 to CT6 (Sidak's multiple-comparisons test, *p* = 0.0027) at a time when neuronal calcium and electrical activity is rising to the peak (Colwell, 2011; Patton et al., 2020). Conversely, treatments given after this time did not show the same acute suppression of the peak (Sidak's multiple-comparisons test: CT6 to CT12, *p* = 0.68; CT12 to CT18, *p* = 0.57; CT18 to CT24, *p* = 0.68). Assessment of the acute effects on cycle amplitude revealed the same pattern (Fig. 5*C*), with CNO suppressing the amplitude of the first cycle post-treatment (repeated-measures two-way ANOVA treatment effect: *F*_(1,52)_ = 8.14, *p* = 0.0062) without revealing a phase-specific or interaction effect (repeated-measures two-way ANOVA phase effect: *F*_(3,52)_ = 0.81, *p* = 0.5; treatment-by-phase interaction: *F*_(3,52)_ = 0.87, *p* = 0.5). Again, *post hoc* tests revealed that this change in peak amplitude manifested as a suppression specifically during early circadian day, CT0 CT6 (Sidak's multiple-comparisons test, *p* = 0.04) without effect at other phases (Sidak's multiple-comparisons test: CT6 to CT12, *p* = 0.29; CT12 to CT18, *p* = 0.9; CT18 to CT24, *p* = 0.96).

We then assessed the sustained effects of neuronal chemogenetic inhibition on ensemble phase. Although the treatment effect did not reach significance (repeated-measures two-way ANOVA treatment effect: *F*_(1,8)_ = 2.91, *p* = 0.13), consistent with the acute effects we saw at CT0 to CT6, there was a significant effect of phase of treatment (repeated-measures two-way ANOVA phase effect: *F*_(3,24)_ = 4.23, *p* = 0.016). However, the interaction between phase and treatment failed to reach significance (repeated-measures two-way ANOVA phase-by-treatment interaction: *F*_(3,12)_ = 3.33, *p* = 0.057). Further examination of the phase-specific effect revealed that, again consistent with the acute effects on amplitude, chemogenetic manipulation during the early circadian day, CT0 to CT6, elicited a significant phase advance (Sidak's multiple-comparisons test, *p* = 0.019), whereas treatment at later phases was without effect (Sidak's multiple-comparisons test: CT6 to CT12, *p* = 0.45; CT12 to CT18, *p* > 0.99; CT18 to CT24, *p* = 0.80).

We then assessed the effects of Gi-activation in astrocytes (Fig. 5*E–H*). The height of the first peak following chemogenetic manipulation was not significantly altered by treatment with CNO (repeated-measures two-way ANOVA treatment effect:& *F*_(1,6)_ = 5.16, *p* = 0.064), but was associated with a phase-dependent effect (repeated-measures two-way ANOVA phase effect, *F*_(3,18)_ = 4.38, *p* = 0.018). Despite this, there was no interaction between phase and treatment (repeated-measures two-way ANOVA phase-by-treatment interaction: *F*_(3,6)_ = 2.23, *p* = 0.19). We therefore investigated the phase-dependent effect further by *post hoc* multiple-comparisons testing, and saw that there was a significant effect of treatment with CNO during the middle of circadian day, CT6 to CT12 (Sidak's multiple-comparisons test, *p* = 0.04), but no effect at any other phase (Sidak's multiple-comparisons test: CT0 to CT6, *p* > 0.99; CT12 to CT18, *p* = 0.27; CT18 to CT24, *p* > 0.99; Fig. 5*F*), leading us to conclude that the phase dependence arises because of a small suppression of the peak amplitude when astrocytes are acutely manipulated during late circadian day, a phase at which their [Ca^2+^]_i_ is rising (Fig. 4*A*; Brancaccio et al., 2017) and at which fluorocitrate treatment is most acutely effective (Fig. 1*H*). This further confirmed efficacy of the Gi-activation in astrocytes, consistent with our calcium data (Fig. 4*D*,*E*). Despite this observation, there was no significant effect of phase (repeated-measures two-way ANOVA phase effect: *F*_(3,18)_ = 2.23, *p* = 0.12), of treatment (repeated-measures two-way ANOVA treatment effect: *F*_(1,6)_ = 4.34, *p* = 0.08), or of interaction (repeated-measures two-way ANOVA phase-by-treatment interaction: *F*_(3,6)_ = 0.93, *p* = 0.48) when we assessed the acute effects on cycle amplitude (Fig. 5*G*).

Having established this weak acute effect of astrocytic Gi-coupled signaling on TTFL waveform, we then determined whether there was a sustained effect on ensemble phase (Fig. 5*H*). This revealed no significant effect of phase (repeated-measures two-way ANOVA phase effect: *F*_(3,18)_ = 2.89, *p* = 0.064), of treatment (repeated-measures two-way ANOVA treatment effect: *F*_(1,6)_ = 3.03, *p* = 0.13), or of interaction between phase and treatment (repeated-measures two-way ANOVA phase-by-treatment effect: *F*_(3,6)_ = 0.22, *p* = 0.88; Fig. 5*H*). These data reveal, therefore, that chemogenetic inhibition of neurons can strongly advance the phase of the ensemble circadian oscillation in a phase-dependent manner, associated with an acute reduction in the amplitude of the following cycle. In contrast, the same manipulation in astrocytes does not induce a phase shift at any phase of the circadian oscillation or acutely alter the waveform following treatment, although it may weakly disrupt the peak following treatment. Thus, neuronal Gi-signaling revealed effective control of SCN phase by neurons, but regulation of Gi signals in astrocytes was without long-term effect.

**Figure 6.**
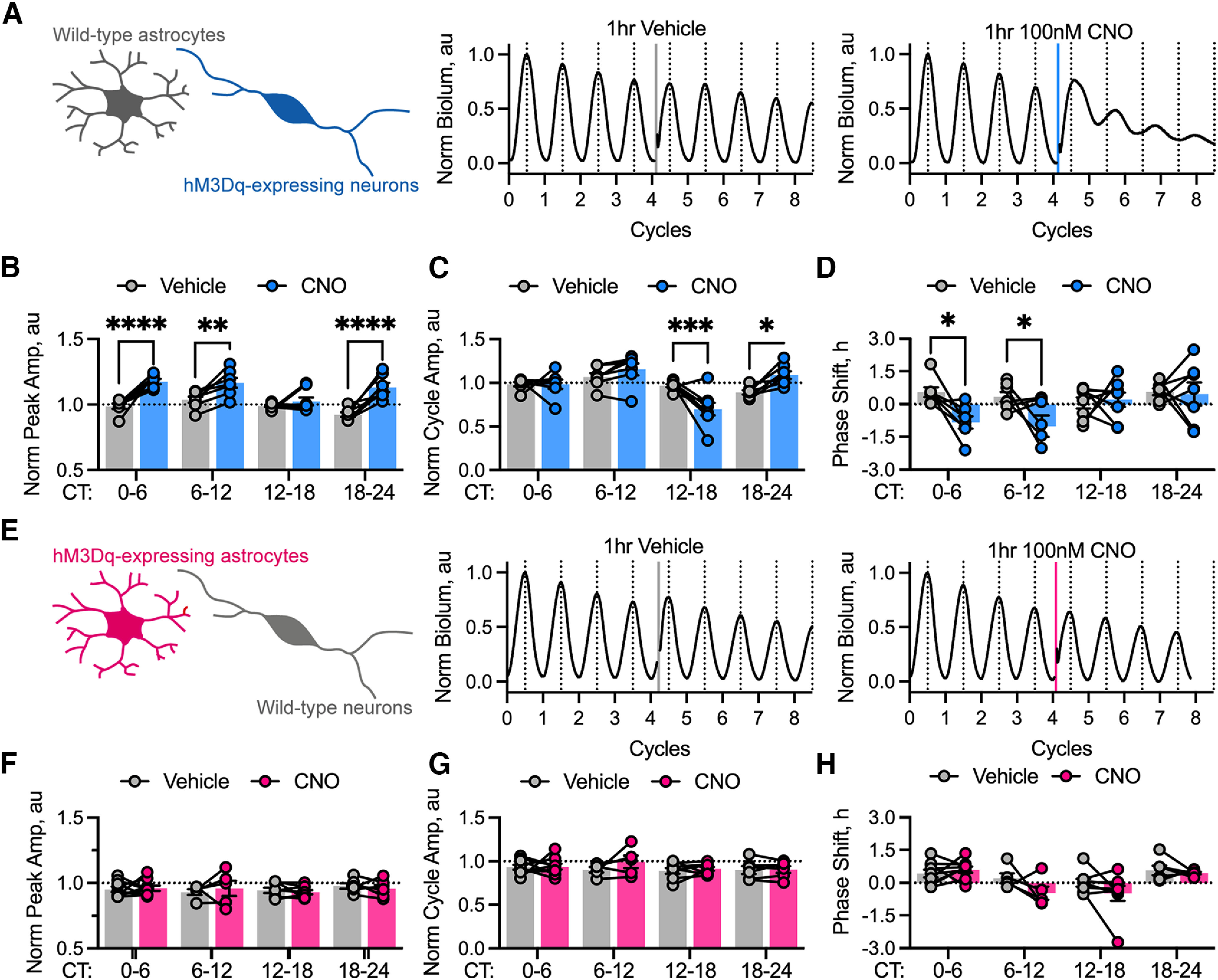
Chemogenetic activation of a Gq-coupled pathway in neurons, but not astrocytes, resets SCN ensemble phase. ***A***, Schematic showing cell type-specific targeting of Gq-coupled DREADDs to neurons (*Syn*-hM3Dq::mCherry), but not astrocytes in the intact SCN network (left) alongside representative PMT traces showing acute vehicle (middle) and 100 nM CNO (right) treatment during the CT0 to CT6 time window. The treatment interval is shown as a vertical line and colored according to treatment: vehicle (gray) or CNO (blue). ***B***, Summary peak amplitude data arranged by phase window of treatment showing paired recordings at that window for SCN with neuronally expressed Gq-coupled DREADDs treated with vehicle (gray, *N* ≥ 7 at each phase) or 100 nM CNO (blue, *N* ≥ 7 at each phase). Statistics: Sidak's multiple-comparisons test, **p = 0.006, ****p < 0.0001. ***C***, Summary cycle amplitude data arranged by phase window of treatment showing paired recordings at that window for SCN with neuronally expressed Gq-coupled DREADDs treated with vehicle (gray, *N* ≥ 7 at each phase) or 100 nM CNO (blue, *N* ≥ 7 at each phase). Statistics: Sidak's multiple-comparisons test, *p = 0.02, ***p = 0.0006. ***D***, Summary phase-shift data arranged by phase window of treatment showing paired recordings at that phase window for SCN with neuronally expressed Gq-coupled DREADDs treated with vehicle (gray, *N* ≥ 7 at each phase) or 100 nM CNO (blue, *N* ≥ 7 at each phase). Statistics: Sidak's multiple-comparisons test, *p ≤ 0.029. ***E***, Schematic showing cell-type-specific targeting of Gq-coupled DREADDs to astrocytes (*GFAP*-hM3Dq::mCherry), but not neurons in the intact SCN network (left) alongside representative PMT traces showing acute vehicle (middle) and 100 nM CNO (right) treatment during the CT0 to CT6 time window. The treatment interval is shown as a vertical line and colored according to treatment: vehicle (gray) or CNO (pink). ***F***, Summary peak amplitude data arranged by phase window of treatment showing paired recordings at that window for SCN with astrocytically expressed Gq-coupled DREADDs treated with vehicle (gray, *N* ≥ 5 at each phase) or 100 nM CNO (pink, *N* ≥ 5 at each phase). ***G***, Summary cycle amplitude data arranged by phase window of treatment showing paired recordings at that window for SCN with astrocytically expressed Gq-coupled DREADDs treated with vehicle (gray, *N* ≥ 5 at each phase) or 100 nM CNO (pink, *N* ≥ 5 at each phase). ***H***, Summary phase-shift data arranged by phase window of treatment showing paired recordings at that phase window for SCN with astrocytically expressed Gq-coupled DREADDs treated with vehicle (gray, *N* ≥ 5 at each phase) or 100 nM CNO (pink, *N* ≥ 5 at each phase). In all histogram plots, individual points joined by lines represent individual slices with paired treatment and histogram bars with error bars represent the mean ± SEM.

We then used Gq-DREADD to activate [Ca^2+^]_i_, first in neurons, recording any acute or sustained effects on the rhythm of Per2::Luciferase activity of SCN slices (Fig. 6*A–D*). The height of the first peak following treatment was enhanced, manifesting as significant effects of phase (repeated-measures two-way ANOVA phase effect: *F*_(3,25)_ = 5.92, *p* = 0.003), of treatment (repeated-measures two-way ANOVA treatment effect: *F*_(1,25)_ = 63.48, *p* < 0.0001), and of an interaction between phase and treatment (repeated-measures two-way ANOVA phase-by-treatment interaction: *F*_(3,25)_ = 5.18, *p* = 0.006). *Post hoc* multiple comparisons revealed that these effects were caused by an acute increase in the height of the first peak after treatment during the circadian day or the late circadian night (Sidak's multiple-comparisons test: CT0 to CT6, *p* < 0.0001; CT6 to CT12, *p* = 0.006; CT18 to CT24, *p* < 0.0001) but not during the early circadian night (Sidak's multiple-comparisons test: CT12 to CT18, *p* = 0.77; Fig. 6*B*). We further examined the acute effects on cycle amplitude following treatment, expecting this to follow suit as with the neuronal Gi-signaling (Fig. 5*B*,*C*). Assessing cycle amplitude, we saw that there was a significant effect of phase (repeated-measures two-way ANOVA phase effect: *F*_(3,25)_ = 10.23, *p* = 0.0001) but no significant effect of treatment (repeated-measures two-way ANOVA treatment effect: *F*_(1,25)_ = 0.02, *p* = 0.88). There was, however, a significant interaction between phase and treatment (repeated-measures two-way ANOVA phase-by-treatment interaction: *F*_(3,25)_ = 10.6, *p* = 0.0001). We therefore investigated the phase-specific effects via *post hoc* multiple comparisons, revealing a complex phenotype whereby cycle amplitude remained unchanged after treatment in circadian day (Sidak's multiple-comparisons test: CT0 to CT6, *p* > 0.99; CT6 to CT12, *p* = 0.54), but was reduced during early circadian night, CT12 to CT18 (*p* = 0.0006), and this increased during late circadian night, CT18 to CT24 (Sidak's multiple-comparisons test, *p* = 0.018). These changes can be attributed to a combination of the previously described peak changes (Fig. 6*B*) and an increased baseline (Fig. 6*A*), a well characterized feature of neuronal Gq-manipulation in SCN slices presumed to be acting through the VIP signaling axis (Brancaccio et al., 2013; Hamnett et al., 2019). The potent acute effects of Gq-activation in neurons were reflected in sustained effects on ensemble phase (Fig. 6*D*), which revealed a significant effect of treatment (repeated-measures two-way ANOVA treatment effect: *F*_(1,50)_ = 8.10, *p* = 0.006). Although there was no overall effect of phase (repeated-measures two-way ANOVA phase effect: *F*_(3,50)_ = 2.40, *p* = 0.08), there was a significant interaction between phase and treatment (repeated-measures two-way ANOVA phase-by-treatment interaction: *F*_(3,50)_ = 2.98, *p* = 0.040). *Post hoc* comparisons revealed a significant phase delay when slices were treated during circadian day (Sidak's multiple-comparisons test: CT0 to CT6, *p* = 0.023; CT6 to CT12, *p* = 0.028) but no phase shift when slices were treated during circadian night (Sidak's multiple-comparisons test: CT12 to CT18, *p* < 0.99; CT18 to CT24, *p* < 0.99). Gq-coupled manipulation of neurons therefore altered TTFL waveform in a phase-dependent manner and strongly reset ensemble phase.

We then assessed the acute and sustained effects of Gq-manipulation in astrocytes (Fig. 6*E*). The amplitude of the first peak of Per2::Luciferase following treatment (Fig. 6*F*) did not show any significant effect of treatment (repeated-measures two-way ANOVA, treatment effect: *F*_(1,9)_ = 0.01, *p* = 0.95), phase (repeated-measures two-way ANOVA phase effect: *F*_(3,27)_ = 0.47, *p* = 0.71), or an interaction between the two (repeated-measures two-way ANOVA, phase-by-treatment interaction: *F*_(3,3)_ = 0.36, *p* = 0.79). We then assessed the acute effects on cycle amplitude, and again there were no significant effects of treatment (repeated-measures two-way ANOVA, treatment effect: *F*_(1,9)_ = 0.63, *p* = 0.44), phase (repeated-measures two-way ANOVA, phase effect: *F*_(3,27)_ = 0.28, *p* = 0.84), or any interaction (repeated-measures two-way ANOVA, phase-by-treatment interaction: *F*_(3,3)_ = 0.66, *p* = 0.63; Fig. 6*G*). Thus, manipulation of astrocytic Gq-signaling pathways did not acutely alter the waveform of SCN ensemble molecular timekeeping. We then assessed whether the direct manipulation of Gq in astrocytes could reset ensemble phase (Fig. 6*H*). This revealed no significant effect of treatment itself (repeated-measures two-way ANOVA, treatment effect: *F*_(1,9)_ = 3.37, *p* = 0.10), although there was a significant effect of phase of treatment (repeated-measures two-way ANOVA, phase effect: *F*_(3,27)_ = 5.39, *p* = 0.005), but no interaction between phase and treatment (repeated-measures two-way ANOVA, phase-by-treatment interaction: *F*_(3,3)_ = 3.12, *p* = 0.19). Multiple *post hoc* comparisons of the phase main effect revealed no significant differences between CNO and vehicle treatment at any phase (Sidak's multiple-comparisons test: CT0 to CT6, *p* > 0.99; CT6 to CT12, *p* = 0.09; CT12 to CT18, *p* = 0.29; CT18 to CT24, *p* = 0.80). We therefore concluded that the phase dependence largely arose from fluctuations because of the experimental protocol and not from a sustained phase shift induced by the activation of Gq in astrocytes. Thus, TTFL phase in the SCN is acutely sensitive to activation of both Gi- and Gq-coupled pathways in neurons, but not in astrocytes. Together, these experiments reveal important differences between the contributions made by astrocytes and neurons in regulating the emergent properties of the SCN circadian network. Whereas ensemble phase is bidirectionally sensitive to neuronal activity, it is not affected by chemogenetic stimulation of astrocytes. In contrast, astrocytes play important roles in maintaining the properties of the ongoing SCN oscillation: its amplitude, intercellular synchrony, and ensemble period.

## Discussion

SCN astrocytes display circadian TTFL and [Ca^2+^]_i_ rhythms in stable antiphase to those of SCN neurons (Brancaccio et al., 2017; Tso et al., 2017; Brancaccio et al., 2019). This implies that strong coupling signals between their respective TTFLs sustain and organize network-wide oscillations, (Hastings et al., 2018). Consistent with this, chronic treatment of SCN slices with the metabolic gliotoxin fluorocitrate, which disrupted astrocytic [Ca^2+^]_i_ oscillations also suppressed the amplitude of the ongoing ensemble neuronal TTFL (Per2::Luciferase) oscillation. Metabolic compromise of astrocytes also impaired cellular synchrony, indicative of looser network coupling, a phenotype more commonly associated with compromised neuronal signaling (Fig. 1; Yamaguchi et al., 2003; Maywood et al., 2006). Importantly, the immediate effect of fluorocitrate was phase dependent, being evident between CT0 to CT6 and CT18, the rising phase of astrocytic activation. Conversely, application during declining astrocytic activity (CT18 to CT0–CT6) saw the effect on the TTFL delayed to the next cycle, consistent with a phase-restricted contribution of astrocytes to network function. This result extends the report that fluorocitrate applied at CT0 to CT6 to acutely prepared rat SCN slices disrupted neuronal electrical activity rhythms on the following cycle (Prosser et al., 1994). Given that SCN firing and TTFL function are intimately linked (Colwell, 2011), it remains to be determined which of them is the primary target of astrocytic signals. Thus, astrocytes reinforce SCN neuronal rhythmicity, potentially through appropriate inhibition of neuronal electrical activity during circadian night when astrocytes are most active. Although the metabolically sensitive astrocytic signals await identification, it may involve astrocytic glutamate release, which is sensed by presynaptic neuronal NR2C receptors, (Brancaccio et al., 2017). Consistent with this model, fluorocitrate inhibits glutamate and glutamine synthesis from radiolabeled glucose in cultured cortical and cerebellar astrocytes (Hassel et al., 1995), while inhibition of NR2C signaling suppresses the amplitude of SCN Per2::Luciferase oscillations, similar to fluorocitrate treatment (Brancaccio et al., 2017). Additionally, astrocyte-mediated active uptake of GABA (Barca-Mayo et al., 2017) or an endocannabinoid/adenosine signaling axis (Hablitz et al., 2020) may also control neuronal rhythms.

The potency of astrocytic signals was most evident in the initiation of rhythmic Per2 expression in Cry-null SCNs (Fig. 2), occurring alongside *de novo* neuronal [Ca^2+^]_i_ rhythms (Brancaccio et al., 2019). The latter may involve calcium-dependent cytosolic signaling pathways impinging on *Per* gene expression (Travnickova-Bendova et al., 2002), and/or CHRONO or DEC1/2 proteins compensating for the absence of Cry proteins (Ono et al., 2021). Such indirect, paracrine astrocytic control of neuronal TTFLs may explain why genetic complementation of Cry in astrocytes takes longer to initiate or amend the network period than does neuronal complementation. During the lag, it is possible that progressive mutual reinforcement between initially weak astrocytic TTFLs (Prolo et al., 2005) and defective Cry-null neuronal clocks leads to an iterative autoamplification of astrocyte-to-neuron and neuron-to-astrocyte signals, slowing building ensemble TTFL amplitude. As SCN neurons are enriched for neuropeptide expression (Morris et al., 2021), these molecules might modulate neuronal feedback to SCN astrocytes. Indeed, the SCN-enriched neuropeptide VIP has been implicated as an entraining and synchronizing factor for astrocytic circadian rhythms (Prolo et al., 2005; Marpegan et al., 2009; Sueviriyapan et al., 2020). Importantly, the end-point oscillations maintained by astrocytes are indistinguishable from neuronally driven oscillations.

Astrocytes, as well as neurons, slowed SCN rhythms to match the period of their cell-autonomous TTFL (Fig. 3), with SCN period reflected at the behavioral level (Brancaccio et al., 2017; Tso et al., 2017; Brancaccio et al., 2019). Again, astrocytes took longer to achieve this (Fig. 3). Nevertheless, Cry1-expressing astrocytes were effective, and, indeed, when SCN astrocytes lack a functional molecular clock, following BMAL1 deletion, they can also slow down the circadian oscillation at the network and the behavioral levels (Barca-Mayo et al., 2017; Tso et al., 2017). A more dramatic difference between neurons and astrocytes came, however, with acceleration of TTFL rhythms by Cry2 complementation of Cry2-null SCNs. Neurons shortened SCN ensemble period by ∼1.5 h, to match their cell-autonomous period, confirming their role as strong pacemakers through bidirectional control of the ongoing oscillation. The effect of accelerating the cell-autonomous period of SCN astrocytes, however, was a marginal shortening of ∼0.4 h. This confirms their bidirectional influence, but also highlights their junior role relative to neurons. Conceptually, slowing down circadian timekeeping (which could be achieved by inhibiting a single rate-limiting process) may be easier than accelerating it, which would require simultaneous regulation of all processes. Thus, indirect, likely inhibitory signals from astrocytes are less effective at the circuit level than is direct manipulation of the neuronal TTFL.

We observed differences between astrocytes and neurons in their cellular response to DREADD activation. Whereas activation of Gi-coupled (hM4Di) or Gq-coupled (hM3Dq) DREADDs achieved inhibition and excitation of neuronal activity, respectively (Roth, 2016), in astrocytes, the activation of either DREADD subtype triggered rises in [Ca^2+^]_i_ (Fig. 4; Yu et al., 2020; Shen et al., 2021), albeit with qualitative differences. Consistent with dorsal striatum and hippocampus (Chai et al., 2017; Durkee et al., 2019; Nagai et al., 2019), Gi-activation in SCN astrocytes caused a small, rapid calcium increase, although somatic localization of the reporter means we cannot exclude larger changes occurring at distal membrane processes (Semyanov et al., 2020). In contrast, Gq-coupled signaling in astrocytes caused large, prolonged rises across the astrocytic network, consistent with responses in the amygdala (Martin-Fernandez et al., 2017) and hippocampus (Chai et al., 2017; Adamsky et al., 2018; Durkee et al., 2019). Despite these rises indicating astrocytic activation under cell type-specific Gq-manipulation, interpretation of the effect on astrocytic activity may be more complex than anticipated: Gq-activation in cortical astrocytes leads to long-term cessation of calcium spiking despite an elevated baseline. This points toward some level of astrocytic silencing (Vaidyanathan et al., 2021). It is therefore imperative to understand how astrocytic activity encodes circadian time, and whether it is calcium spiking, bulk calcium, or both that are responsible for astrocytic information transfer to the rest of the network. Nevertheless, these observations confirmed chemogenetic manipulation as a useful tool to control astrocytic calcium signaling in the SCN (Semyanov et al., 2020).

Chemogenetic activation of SCN neurons caused acute phase-dependent differences in Per2::Luciferase expression: suppression by Gi and elevation by Gq. These were accompanied by the following phase shifts: advances and delays, respectively, and are consistent, respectively, with behaviorally induced nonphotic resetting by inhibition of SCN neural activity (Gribkoff et al., 1998; Maywood et al., 1999; Jones et al., 2015), and photic resetting by retinally mediated activation of SCN neurons (Shearman et al., 1997; Meijer and Schwartz, 2003; Jones et al., 2015). In contrast, direct chemogenetic activation of Gi or Gq in astrocytes did not affect the phase of the ongoing SCN oscillation (Figs. 5, 6). This observation may be at odds with reports that astrocytes can modulate phase advances in early circadian day in SCN explants via a neuron-to-astrocyte signaling pathway where cannabinoid receptors enhance astrocytic adenosine release (Hablitz et al., 2020). It is likely, therefore, that where SCN astrocytes can modulate phase shifts, they actually mediate a neuronal effect, so their direct activation alone, as presented here, is not sufficient to reset ensemble phase. These differences may reflect SCN cellular architecture; the neuronal network receives strong synaptic inputs, for example from the retinal hypothalamic tract, intergeniculate leaflet, and midbrain raphe (Morin and Allen, 2006), allowing it to respond rapidly to afferent entraining signals. Astrocytes, in contrast, do not receive these signals. Rather, they likely receive input from paracrine sources and via their integration within tripartite synapses (Perea et al., 2009), where they modulate and integrate synaptic communication. It is therefore conceivable that SCN astrocytes modulate the response of the SCN network to afferent signals or influence SCN output in intact animals, whereas by performing these experiments in SCN explants the manipulation is without effect. It remains to be determined whether chemogenetic manipulation of astrocytes has any effects *in vivo*.

A final consideration is that SCN neurons outnumber SCN astrocytes by a factor of 3:1 (Guldner, 1983). However, individual astrocytes have been reported to contact up to 100,000 synapses (Halassa et al., 2007), whereas SCN neurons make up to an estimated 1000 synaptic contacts each (Moore and Bernstein, 1989). Despite their lower abundance, therefore, the astrocytic area of pan-network influence may be similar to, or greater than, that of neurons. Furthermore, experiments conditionally targeting SCN neuronal populations have revealed that a similar proportion of neurons, those expressing the VIP receptor VPAC2 (Patton et al., 2020; Morris et al., 2021), cannot set ensemble phase, lengthen period, or initiate *de novo* rhythmicity by Cry1 complementation alone (Patton et al., 2020). In contrast, Cre-conditional BMAL1 ablation targeted to astrocytes (Barca-Mayo et al., 2017; Tso et al., 2017) or AVP neurons (Mieda et al., 2015; Shan et al., 2020) lengthens behavioral period, which only persists in the SCN explant when astrocytes are targeted. Together, these results suggest that at some level of SCN network computation, astrocytes transfer certain types of circadian information more potently than defined neuronal populations. Thus, understanding the cellular roles and architecture of the SCN astrocyte–neuron network is paramount in understanding how the SCN generates and disseminates time-of-day information.
